# A TLR9 agonist promotes IL-22-dependent pancreatic islet allograft survival in type 1 diabetic mice

**DOI:** 10.1038/ncomms13896

**Published:** 2016-12-16

**Authors:** Deepak Tripathi, Sambasivan Venkatasubramanian, Satyanarayana S. Cheekatla, Padmaja Paidipally, Elwyn Welch, Amy R. Tvinnereim, Ramakrishna Vankayalapati

**Affiliations:** 1 Department of Pulmonary Immunology, Center for Biomedical Research, University of Texas Health Science Center at Tyler, Tyler, Texas 75708, USA

## Abstract

Pancreatic islet transplantation is a promising potential cure for type 1 diabetes (T1D). Islet allografts can survive long term in the liver parenchyma. Here we show that liver NK1.1^+^ cells induce allograft tolerance in a T1D mouse model. The tolerogenic effects of NK1.1^+^ cells are mediated through IL-22 production, which enhances allograft survival and increases insulin secretion. Increased expression of NKG2A by liver NK1.1^+^ cells in islet allograft-transplanted mice is involved in the production of IL-22 and in the reduced inflammatory response to allografts. Vaccination of T1D mice with a CpG oligonucleotide TLR9 agonist (ODN 1585) enhances expansion of IL-22-producing CD3-NK1.1^+^ cells in the liver and prolongs allograft survival. Our study identifies a role for liver NK1.1^+^ cells, IL-22 and CpG oligonucleotides in the induction of tolerance to islet allografts in the liver parenchyma.

## Abstract (web summary)

Tolerance is required to prevent rejection of intrahepatic islet allografts as a potential treatment for type 1 diabetes. Here the authors show that IL-22 produced by NK1.1^+^ cells in the liver of streptozotocin T1D model mice can drive tolerance to allografted islets.

Type 1 diabetes (T1D) is an autoimmune disease that occurs because of the destruction or loss of pancreatic β-cells^1^. Inflammatory immune cells have a major role in destroying pancreatic β-cells^2^. Immunosuppressive drugs and co-stimulatory blockade can prevent inflammation and protect β-cells, but they can also lead to opportunistic infections and cancers^3^,^4^. The long-term effects of these therapies are unknown. Exogenous insulin and intensive insulin therapy can delay diabetes complications, but they can also cause life-threatening hypoglycemia^5^. Pancreatic islet transplantation holds much promise for the cure of T1D because islet grafts can produce physiological insulin and require limited use of immunosuppressive drugs^6^.

Pancreatic islet transplantation into the kidney capsule, testis or liver can protect islets from inflammation because of the immunosuppressive environment in these organs compared with other organs^7^. Islet allografts can survive long term in the liver parenchyma^8^. The liver metabolizes gut-derived foreign antigens, and has a high tolerogenic capacity that favors the induction of peripheral tolerance^9^. In mice, the liver can induce spontaneous tolerance to allografts without any requirement for immune-suppression^10^; however, the mechanisms involved in liver-induced spontaneous tolerance are not well understood. Immune cell populations in the liver, such as dendritic cells and CD4+CD25+Foxp3+ regulatory T (Treg) cells, and costimulatory molecules are important for the induction of tolerance^11^,^12^.

Liver resident natural killer (NK) cells constitute a large proportion of the lymphocyte population^13^, and the liver contains a large population of functionally hypo-responsive NK cells that exhibit unique repertoires and cytokine profiles^14^,^20^. Peripheral and splenic NK cells are generally considered to be pro-inflammatory, and kill target cells without prior antigen priming^15^. However, compared with splenic NK cells, liver NK cells have a weaker IFN-γ response^13^. Liver NK cells express high levels of the inhibitory receptor NKG2A and lack expression of MHC class I-binding Ly49 receptors^16^. Adoptively transferred splenic NK cells that migrate to the liver display phenotypic and functional changes^14^, supporting the view that the liver environment modifies NK cell receptor expression and functional responsiveness. These qualities of liver NK cells suggest they can induce tolerance to allografts. Previous studies using a skin transplant model have shown that peripheral NK cells promote allograft survival by killing donor antigen presenting cells (APCs)^17^. In addition, Beilke *et al*. showed that treatment with a depleting anti-NK1.1 antibody before transplantation induces allograft rejection in mice, suggesting a role for NK cells in tolerance^18^.

In this study, we investigated the role of hepatic NK1.1^+^ cells in pancreatic islet allograft survival in the liver parenchyma. We found that liver NK1.1^+^ cells produce IL-22, which enhances islet allograft survival. IL-22 enhances the expression of islet survival genes and increases insulin production. We also found that liver NK1.1^+^ cells inhibited inflammatory responses to islet allografts through the inhibitory receptor NKG2A. Furthermore, our study demonstrates that the immunization and challenge of T1D mice with a TLR9 agonist can enhance IL-22 production by NK1.1^+^ cells and prolong islet allograft survival. Our study demonstrates that liver NK1.1^+^ cells produce IL-22 to enhance pancreatic islet allograft survival and increase insulin production. The NK cell inhibitory receptor NKG2A plays a role in inhibiting inflammation in response to pancreatic islet allografts, and IL-22 production and ;TLR-9 agonists can be used as therapeutic agents to induce liver NK1.1^+^ cell-mediated tolerance to islet allograft.

## Results

### Liver NK1.1^+^ cells are essential for allograft survival

**Figure 1:**
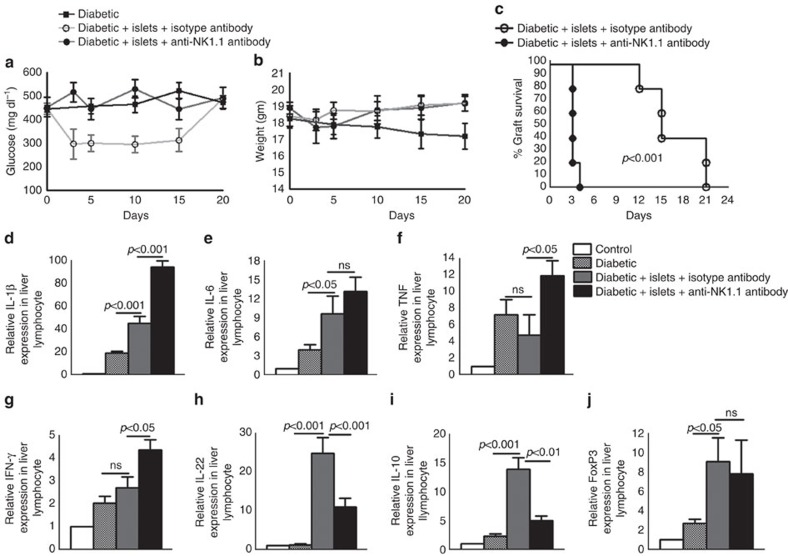
NK1.1 cells enhance islet allograft survival in T1D mice. A single intraperitoneal injection of streptozotocin (STZ) (180 mg kg^−1^ body weight) induced type 1 diabetes (T1D) in C57BL/6 mice as measured by random blood sugar levels after one week. Approximately 200 islets obtained from BALB/c or C57BL/6 mice (donor) were transplanted into the liver parenchyma of T1D C57BL/6 mice (recipients). Some of the islet allograft recipient mice were treated with anti-NK1.1 or isotype control antibodies (0.5 mg per mouse 24 h before and 0 and 24 h after transplantation via tail vein injection). (**a**) Blood glucose levels were measured every 72 h until 24 days. Blood glucose levels above 300 mg dl^−1^ were considered to indicate diabetes or failed glucose control. (**b**) Body weight. (**c**) Percentage graft survival. *P* value for per cent graft survival was calculated using a log rank test. Kaplan–Meier survival curves of mice are shown. (**d**–**j**) Islets from BALB/c mice were transplanted into T1D C57BL/6 mice that were treated with anti-NK1.1 and isotype control antibodies, as mentioned in (**a**). After 3 days, liver lymphocytes were isolated. Cytokine mRNA expression was determined by real-time PCR. Bar graphs show the means±s.d. *P* values were generated by one-way analysis of variance (ANOVA). The data presented are representative of five independent experiments, and five mice per group were used.

We determined the role of liver NK1.1 cells in allograft tolerance in a pancreatic islet transplantation model. First, we induced T1D in C57BL/6 mice as described in the Methods section. Immunohistochemistry of the pancreas from control and T1D C57BL/6 mice is shown in Supplementary Fig. 1. We next isolated pancreatic islets from BALB/c or C57BL/6 mice and transplanted 200 islets into the liver parenchyma of T1D C57BL/6 mice, as described in the Methods section. Some of the islet-transplanted T1D mice also received isotype control or anti-NK1.1 antibodies one day before and one day after the transplantation via an intravenous injection. On the day of transplantation, mice received antibodies along with the islets. We measured non-fasting blood glucose every third day and considered a graft rejected if the glucose levels were >300 mg dl^−1^ (Fig. 1a). The allogeneic islet grafts survived up to 15 days in the mice that received isotype control antibody (*p*<0.001, log-rank test; Fig. 1c). In contrast, the allogeneic islet grafts were rejected within three days in the mice that received anti-NK1.1 antibodies (*p*<0.001, log-rank test; Fig. 1c). The allogeneic islet-transplanted mice that received anti-NK1.1 antibodies also lost weight compared with the mice that received isotype control antibodies (Fig. 1b). As a control, we determined syngeneic islet graft survival in T1D mice that received anti-NK1.1 or isotype control antibodies. We followed these mice up to 40 days, and the grafts were not rejected (Supplementary Fig. 2). These results demonstrate that liver NK1.1 cells are essential for islet allograft survival.

### Liver NK1.1^+^ cells inhibit inflammation to allograft

We determined the mechanisms involved in the induction of NK1.1 cell-mediated tolerance to islet allografts. T1D mice were transplanted with pancreatic islets and treated with isotype or NK1.1 antibodies, as mentioned in Fig. 1a and in the Methods section. Three days after transplantation, liver lymphocytes were isolated, and the gene expression profiles were determined by real-time PCR. As shown in Fig. 1d–j, treatment of islet-transplanted mice with anti-NK1.1 antibody enhanced IL-1β (45.13±5.9 versus 94.25±5.5; *p*<0.001, ANOVA test), TNF (4.746±2.4 versus 11.92±1.8; *p*<0.05, ANOVA test) and IFN-γ (2.714±0.45 versus 4.344±0.4; *p*<0.05, ANOVA test) expression in recipient mouse liver cells compared with treatment with isotype control antibodies. In contrast, treatment with the anti-NK1.1 antibody inhibited IL-10 (13.95±1.9 versus 5.03±0.83; *p*<0.01, ANOVA test) and IL-22 (24.82±4.1 versus 11.01±2.2; *p*<0.001, ANOVA test) expression compared with treatment with the isotype antibody. Treatment with anti-NK1.1 antibodies had no effect on the expression of Foxp3 (Fig. 1j). These findings suggest that liver NK1.1 cells inhibit inflammatory cytokine production in response to islet allograft in the liver.

### Liver NK1.1^+^ cells produce IL-22 in response to allograft

**Figure 2:**
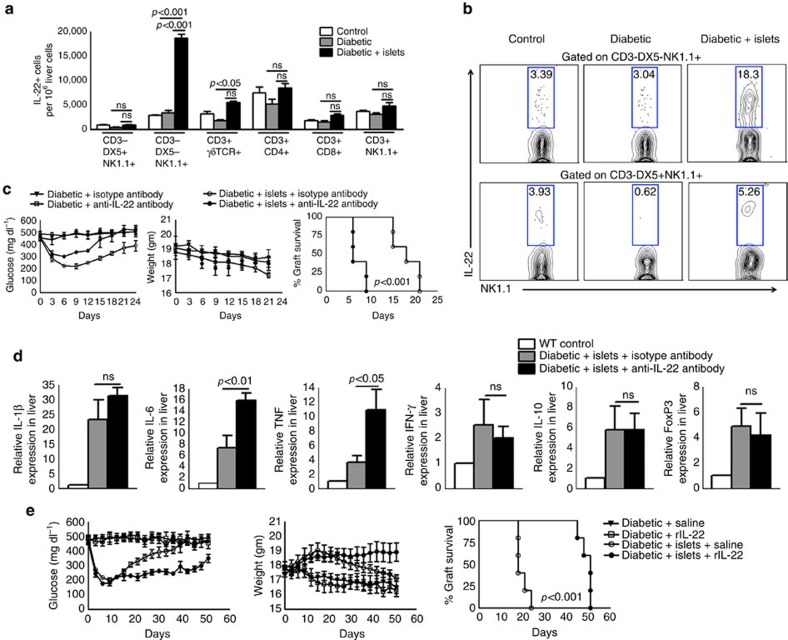
IL-22 produced by liver NK 1.1 cells prolongs islet allograft survival. (**a**) IL-22 production by liver cells. Islets from BALB/c mice were transplanted into T1D C57BL/6 mice as mentioned in Fig. 1. Three days after transplantation, the percentages of IL-22+ cells in the recipient liver were determined by flow cytometry. Bar graphs show the means±s.d. *P* values were generated by one-way analysis of variance (ANOVA). (**b**) A representative flow cytometry plot is shown. (**c**) Anti-IL-22 antibody inhibited islet allograft survival. Islets from BALB/c mice were transplanted into T1D C57BL/6 mice, as shown in Fig. 1. Some of the T1D and islet allograft-transplanted T1D recipient mice were treated with anti-IL-22 or isotype control antibodies (0.3 mg per mouse 24 h before, 0 and 24 h after transplantation through tail vein injection). The *P* value for per cent graft survival was calculated using the log rank test. The Kaplan–Meier survival curves of mice are shown. (**d**) Anti-IL-22 antibody enhanced TNF and IL-6 expression. T1D mice were transplanted and treated with anti-IL-22 antibodies, as mentioned in (**c**). After 5 days, liver lymphocytes were isolated, and cytokine mRNA expression levels were determined by real-time PCR. Bar graphs show the means±s.d. *P* values were generated by one-way analysis of variance (ANOVA). (**e**) Recombinant IL-22 treatment prolonged allograft survival. T1D and islet transplanted-TID mice were treated with saline or recombinant IL-22 (100 ng kg^−1^ of body weight) starting on day 12 and continuing twice weekly for 50 days. As a control, some of the TID mice (not islet-transplanted) were also treated with saline or rIL-22 (100 ng kg^−1^ of body weight). The *P* value for per cent graft survival was calculated using the log rank test. The Kaplan–Meier survival curves of mice are shown. The data presented are representative of five independent experiments, and five mice per group were used.

In the above experiment, treatment of pancreatic islet allograft recipient mice with anti-NK1.1 antibodies reduced IL-22 expression by 2.2-fold compared with the isotype control antibody. IL-22 has been associated with liver tissue repair and/or regeneration in murine models of microbial infection^19^. To determine the source of IL-22 in pancreatic islet allograft recipient mice, T1D mice were transplanted with pancreatic islets, as mentioned in Fig. 1. Three days after transplantation, flow cytometric analysis of liver cells indicated an increased number of IL-22-producing CD3-DX5-NK1.1^+^ cells compared with wild type control (2900±200 versus 18700±1900; *p*<0.001, ANOVA test; Fig. 2a) and T1D mice (3400±1200 versus 18700±1900; *p*<0.001, ANOVA test). γδ-positive liver T cells also produced IL-22, but the absolute number of IL-22-producing CD3-DX5-NK1.1^+^ cells was significantly higher (5500±700 versus 18700±1900; *p*<0.001, ANOVA test) (Fig. 2a; Supplementary Fig. 3b). Other liver lymphocytes, including CD4+ and CD8+ T cells, were not the source of IL-22 (Fig. 2a; Supplementary Fig. 3b).

### IL-22 is essential for allograft survival in T1D mice

T1D mice were transplanted with pancreatic islets, as in Fig. 1. Some of the islet-transplanted T1D mice also received isotype control or anti-IL-22 (0.3 mg per mouse) antibodies one day before and one day after transplantation via an intravenous injection. On the day of transplantation, mice received antibodies along with the islets. The islet grafts survived for 18 days in the mice that received isotype control antibodies (*p*<0.001, log-rank test). In contrast, the islet grafts were rejected within 6 days in the mice that received anti-IL-22 antibodies (*p*<0.001, log-rank test; Fig. 2c). Anti-IL-22 antibody treatment of islet-transplanted mice inhibited the insulin production by islets in the liver parenchyma (Supplementary Fig. 4a). As shown in Fig. 2c, treatment of TID mice with the anti-IL-22 antibody (without islet transplantation) had no effect on blood glucose levels. These results demonstrate that IL-22 is essential for islet allograft survival. We also determined the effects of anti-IL-22 antibody treatment on pro- and anti-inflammatory cytokine gene expression in the liver of islet-transplanted mice. Three days after isotype or anti-IL-22 antibody treatment, mouse liver cells were isolated, and pro- and anti-inflammatory cytokine gene expression was analysed, as described in the Methods section. As shown in Fig. 2d, only the expression of IL-6 (15.75±1.35 versus 7.192±2.20; *p*<0.01, ANOVA test) and TNF (10.80±2.86 versus 3.616±0.86; *p*<0.05, ANOVA test) was up regulated in the anti-IL-22 antibody-treated pancreatic islet-transplanted T1D mice.

We also determined the effect of recombinant IL-22 on allograft survival. T1D mice were transplanted with pancreatic islets, as in Fig. 1. Some of the islet-transplanted T1D mice also received recombinant IL-22 (100 ng kg^−1^ body weight twice weekly via intravenous injection) beginning day 12 post-transplantation and continuing for up to 50 days post-transplantation. The islet grafts survived for 18 days (*p*<0.001, log-rank test) in T1D mice. Recombinant IL-22 injection prolonged graft survival up to 51 days (*p*<0.001, log-rank test; Fig. 2e), and prolonged insulin secretion by the islet allograft in the liver parenchyma (Supplementary Fig. 4b). Recombinant IL-22 treatment of T1D mice (without islet transplantation) had no effect on glucose levels (Fig. 2e).

### IL-22 enhances the survival and insulin production of islets

**Figure 3:**
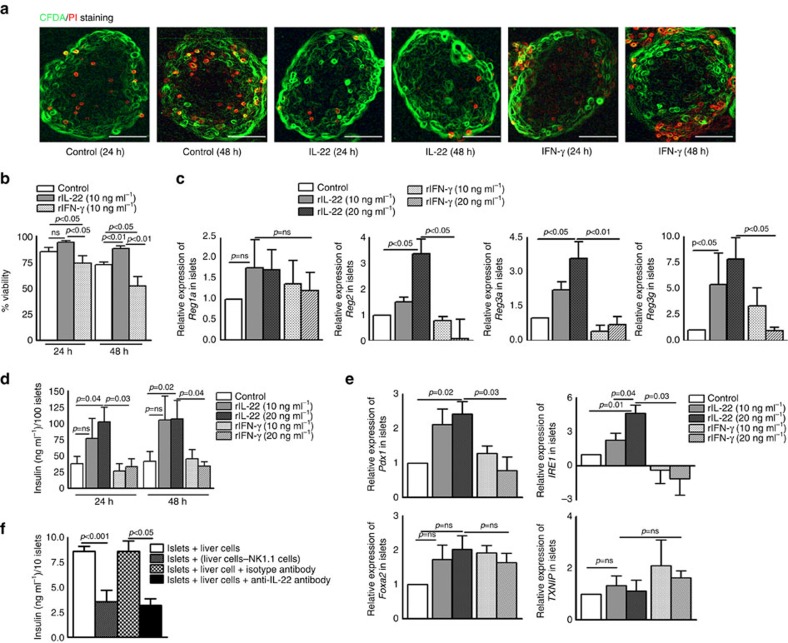
IL-22 produced by NK 1.1 cells enhances survival and insulin production by islets. (**a**,**b**) BALB/c mouse islets (one hundred islets) were cultured in the presence of recombinant IL-22 or IFN-γ (10 and 20 ng ml^−1^) for 24 and 48 h. The islets were labelled with CFDA/PI (carboxyfluorescein diacetate succinimidyl ester/propidium iodide) staining. A fluorescence microscopy image is shown. Photographs are representative of staining patterns. Magnification × 40; scale bar, 50 μm (Right). The percentage of viable islets was determined using a fluorescence microscope. Bar graphs show the means±s.d. *P* values were generated by one-way analysis of variance (ANOVA). (**c**) Islets from BALB/c mice were cultured in the presence of recombinant IL-22 and IFN-γ (10 and 20 ng ml^−1^). The mRNA expression of islet regenerating genes (*Reg2, Reg3a, Reg1a and Reg3g*) was determined by real time PCR. Bar graphs show the means±s.d. *P* values were generated by one-way analysis of variance (ANOVA). (**d**) Insulin levels in the culture supernatants were measured by ELISA. Bar graphs show the means±s.d. *P* values were generated by one-way analysis of variance (ANOVA). (**e**) The mRNA expression of regulatory genes for insulin biosynthesis and secretion (*pdx1, foxa2, IRE1, TXNIP1*) was determined by real time PCR. Bar graphs show the means±s.d. *P* values were generated by one-way analysis of variance (ANOVA). (**f**) Whole liver cells from C57BL/6 mice were cultured with BALB/c mouse islets at a ratio of 10,000:1 (1 × 10^5^ liver cells and 10 islets) in the presence of anti-IL-22 or isotype control IgG2 antibodies (1.5 μg ml^−1^). In some cultures, NK cells were depleted from whole liver cells by positive selection before the liver cells were cultured with the islets. After 24 h, insulin levels in the culture supernatant were measured by ELISA. Bar graphs show the means±s.d. *P* values were generated by one-way analysis of variance (ANOVA). The data from four independent experiments are shown.

As shown in Fig. 2c, anti-IL-22 antibodies inhibited pancreatic islet allograft survival in T1D mice. However, anti-IL-22 had only a marginal effect on pro-inflammatory cytokine production, suggesting that IL-22 may be regulating graft survival by other mechanisms. Pancreatic islets express the IL-22 receptor (Supplementary Fig. 5), and IL-22 can protect islets from oxidative and endoplasmic reticulum (ER) stress in response to inflammatory cytokines in diabetic mice^21^. Next, we investigated the effects of IL-22 on the survival of islets and on insulin production. Pancreatic islets were cultured in the presence of recombinant IL-22 or IFN-γ (10 ng ml^−1^) for 24 and 48 h, and the viability of the islets was determined by fluorescence microscopy. In four independent experiments, recombinant IL-22 significantly enhanced the survival of islets compared with the islets cultured in media alone for 48 h (72.50±2.50 versus 87.99±2.5; *p*<0.05, ANOVA test; Fig. 3b). In contrast, recombinant IFN-γ disintegrated the islets, and the islet viability in this group was only 51%. The Regenerating (*Reg*) genes *Reg2, Reg3a* and *Reg3g* are known to enhance pancreatic islet survival. We analysed the expression of these genes in cultured cells. As shown in Fig. 3c, recombinant IL-22 induced 3-fold more *Reg2* (*p*<0.05, ANOVA test), 1.7-fold more *Reg3a* (*p*<0.05, ANOVA test) and 7.4-fold more *Reg3g* (*p*<0.05, ANOVA test) expression compared with islets cultured with media. In contrast, recombinant IFN-γ had no effect on *Reg2, Reg3a* or *Reg3g* expression.

We also measured insulin levels in the above described culture supernatants. Recombinant IL-22 enhanced insulin production from 41.61±14.85 to 102.0±22.65 ng ml^−1^, *p*=0.04, ANOVA test. In contrast, recombinant IFN-γ had no effect on insulin production (41.61±14.85 versus 33.41±12.07; *p*=0.68, ANOVA test; Fig. 3d). As shown in Fig. 3d, recombinant IL-22 enhanced insulin levels in a concentration-dependent manner. Pancreatic and duodenal homeobox 1 (*Pdx1*) and Inositol-Requiring Enzyme 1 (*IRE1*) are known to induce insulin production in pancreatic islets. As shown in Fig. 3e, incubation with recombinant IL-22 resulted in 2.3-fold more *Pdx1* (*p*=0.02, ANOVA test) and 4.6-fold more *IRE1* (*p*=0.01, ANOVA test) expression compared with islets cultured with media alone. In contrast, recombinant IFN-γ had no effect on *Pdx1* or *IRE1* expression.

We next determined the effects of IL-22 on insulin production in a more physiological setting. We isolated liver cells from T1D C57BL/6 mice; 1 × 10^5^ whole liver cells or NK1.1 cell-depleted liver (NKDL) cells (depleted as mentioned in the Methods) were cultured with 10 pancreatic islets obtained from BALB/c mice. Some of the islets cultured with whole liver cells were also cultured with anti-IL-22 antibodies or isotype antibodies. After three days, we measured insulin levels in the culture supernatants by ELISA. Culturing the T1D C57BL/6 mouse whole liver cells with pancreatic islets of BALB/c mice induced 8.7±0.8 ng ml^−1^ of insulin. Insulin levels were reduced to 3.2±1.1 ng ml^−1^ (*p*<0.001, ANOVA test; Fig. 3f) by the addition of anti-IL-22 antibodies. Depletion of NK1.1 cells from whole liver cells before culturing with islets also reduced insulin levels to 3.5±1.9 ng ml^−1^ (*p*<0.001, Fig. 3f).

### Liver CD3-DX5-NK1.1^+^ cells prolong islet allograft survival

**Figure 4:**
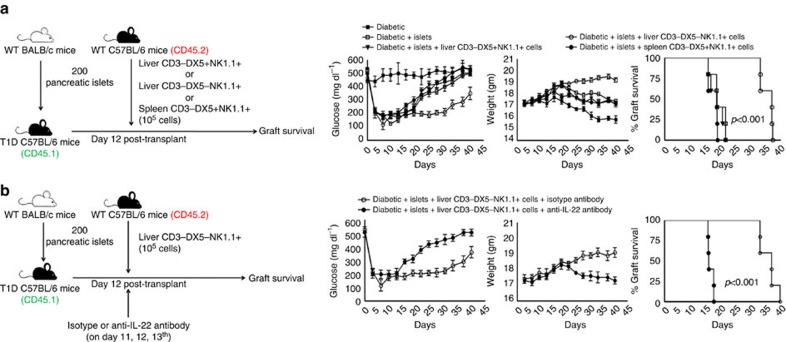
Liver CD3-DX5-NK1.1^+^ cells prolong islet allograft survival. (**a**) Islets from BALB/c mice were transplanted into the liver parenchyma of CD45.1 T1D C57BL/6 mice (recipients) as mentioned in Fig. 1. Twelve days after transplantation, 10^5^ liver CD3-DX5-NK1.1^+^ or CD3-DX5+NK1.1^+^ or splenic CD3-DX5+NK1.1^+^ cells from CD45.2 C57BL/6 mice were adoptively transferred via tail vein injection (recipient CD45.1 T1D C57BL/6 mice). Blood glucose and body weight were measured every 72 h until 40 days. The P value for per cent graft survival was calculated using the log rank test. Kaplan–Meier survival curves of mice are shown. (**b**) IL-22 produced by the liver CD3-DX5-NK1.1^+^ cells prolongs islet allograft survival. Islets from BALB/c mice were transplanted into the liver parenchyma of CD45.1 T1D C57BL/6 mice (recipients), as mentioned in Figs 1 and 2c. Twelve days after transplantation, 10^5^ liver CD3-DX5-NK1.1^+^ cells from CD45.2 C57BL/6 mice were adoptively transferred along with the anti-IL-22 or isotype control antibodies (0.3 mg per mouse on day 12, 13 and 14 through tail vein injection). Blood glucose and body weight were measured every 72 h until 40 days. The *P* value for per cent graft survival was calculated using the log rank test. Kaplan–Meier survival curves of mice are shown. The data presented are representative of five independent experiments. In all mouse experiments, five mice were used per group.

The above findings demonstrated that NK1.1 cells produce IL-22 and that IL-22 prolongs islet allograft survival in the liver parenchyma. We determined whether IL-22-producing liver resident NK1.1 cells are responsible for islet allograft survival. First, we isolated liver CD3-DX5-NK1.1^+^, liver CD3-DX5+NK1.1^+^ and splenic CD3-DX5+NK1.1^+^ cells from CD45.2 mice (C57BL/6), as mentioned in the Methods. Liver CD3-DX5-NK1.1^+^, liver CD3-DX5+NK1.1^+^ or splenic CD3-DX5+NK1.1^+^ cells (10^5^) were adoptively transferred into CD45.1 T1D mice by tail vein injection on the 12th day after islet allograft transplantation. As shown in Supplementary Fig. 6a, a significant percentage of adoptively transferred cells were detected in the liver after 6 days. Islet allografts survived up to 35 days in the mice that received liver CD3-DX5-NK1.1^+^ cells (Fig. 4a). In contrast, islet allografts were rejected within 2–6 days in the mice that received CD3-DX5+NK1.1^+^ or splenic CD3-DX5+NK1.1^+^ cells (Fig. 4a). Seven days after adoptive transfer, the frequency of IL-22-producing donor-derived CD3-DX5-NK1.1^+^ (CD45.2) cells was significantly higher compared with CD3-DX5+NK1.1^+^ cells in the recipient liver (35.4±4.8 versus 10.8±2.7; p<0.01, *t*-test; Supplementary Fig. 6b). Next, we investigated whether IL-22 produced by donor-derived CD3-DX5-NK1.1^+^ cells prolongs islet allograft survival. We adoptively transferred liver CD3-DX5-NK1.1^+^ cells from CD45.2 mice to CD45.1 T1D mice along with anti-IL-22 or isotype control antibodies on the 12th day after islet allograft transplantation. Islet allografts survived up to 4 days in the mice that received the CD3-DX5-NK1.1^+^ cells along with anti-IL-22 antibody. In contrast, islet allografts survived up to 37 days in the mice that received CD3-DX5-NK1.1^+^ cells along with isotype antibody (*p*<0.001, log-rank test; Fig. 4b).

We further determined insulin production by islets in the presence of liver CD3-DX5-NK1.1^+^ or CD3-DX5+NK1.1^+^ or splenic CD3-DX5+NK1.1^+^ cells. Pancreatic islets from BALB/c mice were cultured with the whole liver cells or NKDL cells from T1D C57BL/6 mice, as mentioned in the methods section. Some of the NKDL cultured islets were cultured with liver CD3-DX5-NK1.1^+^ or CD3-DX5+NK1.1^+^ or splenic CD3-DX5+NK1.1^+^ cells, and insulin levels in the culture supernatants were measured after 72 h. Insulin levels in the culture supernatants were 7.6±2.4 (ng ml^−1^), when islets were cultured with whole liver cells. This was reduced to 3.4±0.9 (ng ml^−1^) on culturing with NKDL cells. The addition of liver CD3-DX5-NK1.1^+^ cells but not liver CD3-DX5+NK1.1^+^ or splenic CD3-DX5+NK1.1^+^ cells to islets cultured with NKDL cells increased insulin production to 6.1±0.7 (ng ml^−1^) (Supplementary Fig. 6c). In addition, we found increased IL-22 levels in the culture supernatants of liver CD3-DX5-NK1.1^+^ cells, but not liver CD3-DX5+NK1.1^+^ or splenic CD3-DX5+NK1.1^+^ cells added NKDL cultured islets (*p*<0.001, ANOVA test; Supplementary Fig. 6c). Treating CD3-DX5-NK1.1^+^ cells with the IL-22 siRNA, but not control siRNA before culturing with NKDL cells and islets unable to enhance insulin production (*p*<0.05, ANOVA test; Supplementary Fig. 6d).

### NKG2A inhibits the alloimmune response

**Figure 5:**
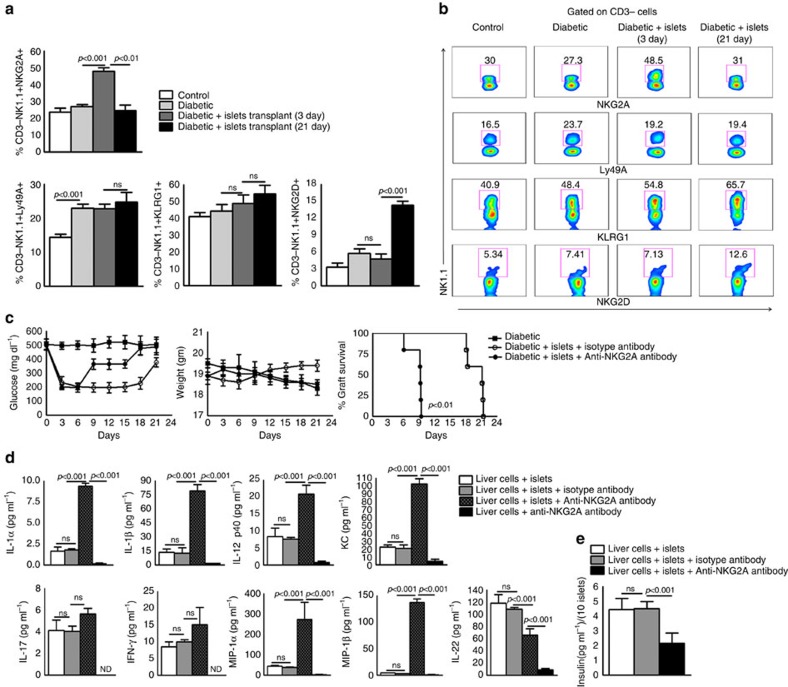
NKG2A expressed by NK1.1 cells inhibits the alloimmune response. (**a**) The expression of NK cell activating and inhibitory receptors. Islets from BALB/c mice were transplanted into T1D C57BL/6 mice, as mentioned in Fig. 1. After 3 and 21 days, the expression of Ly49A, NKG2A, KLRG1 and NKG2D by liver CD3-NK1.1^+^ cells was determined by flow cytometry. Bar graphs show the means±s.d. *P* values were generated by one-way analysis of variance (ANOVA). (**b**) A representative flow cytometry figure is shown. (**c**) Blocking of NKG2A receptor signaling reduces islet allograft survival. Islets from BALB/c mice were transplanted into the liver parenchyma of T1D C57BL/6 mice (recipients) as shown in Fig. 1. Some of the islet allograft recipient mice were treated with anti-NKG2A or isotype control antibodies (0.3 mg per mouse 24 h before, 0 and 24 h after transplantation through tail vein injection). Blood glucose and body weight were measured every 72 h until 24 days. *P* value for per cent graft survival was calculated using a log rank test. Kaplan–Meier survival curves of mice are shown. The data presented are representative of five independent studies. Five mice per group were use. (**d**) Liver cells from T1D mice were isolated and cultured with BALB/c mouse pancreatic islets at a ratio of 10,000:1 (1 × 10^5^:10) in the presence of anti-NKG2A, or isotype control IgG2 antibodies (1.5 μg ml^−1^). After 72 h, various cytokine levels and (**e**) insulin levels were measured by ELISA. Bar graphs show the means±s.d. *P* values were generated by one-way analysis of variance (ANOVA). The data from three independent experiments are shown.

In Fig. 1, we found that liver NK1.1 cells inhibited pro-inflammatory cytokine expression in response to islet allografts and that inhibition was not because of IL-22 (Fig. 2d). We first investigated the potential involvement of NK cell receptors in inhibiting pro-inflammatory immune responses in islet-recipient T1D mice. T1D mice were transplanted with pancreatic islets, as mentioned in Fig. 1 and the methods section. Liver lymphocytes from pancreatic islet-recipient T1D and control T1D mice were isolated 3 days and 21 days after transplantation, as mentioned in the methods section. Expression of the activating receptor NKG2D and of the inhibitory receptors KLRG1, Ly49A and NKG2A on liver CD3-NK1.1^+^ cells was determined by flow cytometry, as mentioned in the methods section. As shown in Fig. 5a,b, three days after pancreatic islet allograft transplantation, the percentage of CD3-NK1.1^+^NKG2A+ cells in recipient T1D mice were increased compared with control T1D mice (27.18±1.09 versus 47.93±2.15; *p*<0.001, ANOVA test). As shown in Fig. 5a,b, 21 days after islet allograft transplantation, the percentage of CD3-NK1.1^+^NKG2D+ cells was increased compared with the third day after transplantation (4.82±0.85 versus 14.30±0.78, *p*<0.001, ANOVA test). We determined the role of NKG2A on islet allograft survival. Some of the islet-transplanted T1D mice also received isotype control or anti-NKG2A antibodies (0.3 mg per mouse) via intravenous injection one day before islet transplantation, on the day of islet transplantation and one day after the transplantation^22^,^23^. The islet allografts survived for 19 days in the mice that received isotype control antibodies. In contrast, islet allografts were rejected within 8 days in the mice that received anti-NKG2A antibodies (*p*<0.001, log-rank test; Fig. 5c).

We next determined the role of NKG2A on the inhibition of pro-inflammatory cytokines. We isolated liver cells from T1D C57BL/6 mice, and 1 × 10^5^ whole liver cells were cultured with 10 pancreatic islets obtained from BALB/c mice. Some of the cells were cultured with anti-NKG2A antibodies or isotype control antibodies, and after three days, chemokine and cytokine levels were measured by multiplex ELISA. As shown in Fig. 5d, culturing T1D C57BL/6 mouse liver cells with pancreatic islets obtained from BALB/c mice significantly enhanced the levels of various pro-inflammatory chemokine and cytokine levels. These levels were further enhanced by blocking the NKG2A receptor. Surprisingly, anti-NKG2A antibody reduced IL-22 production by T1D mouse liver cells (108.29±5.05 to 66.05±18.15; *p*<0.001, ANOVA test; Fig. 5d). Insulin secretion was also decreased from 5.81±0.30 to 2.96±0.26 (*p*<0.001, ANOVA test; Fig. 5e). Silencing of NKG2A or Qa-1b (NKG2A ligand) expression in whole liver cells and culturing them with BALB/c mouse pancreatic islets enhanced inflammatory cytokine and chemokine production (Supplementary Fig. 7).

### Immunization with a TLR9 agonist prolongs allograft survival

**Figure 6:**
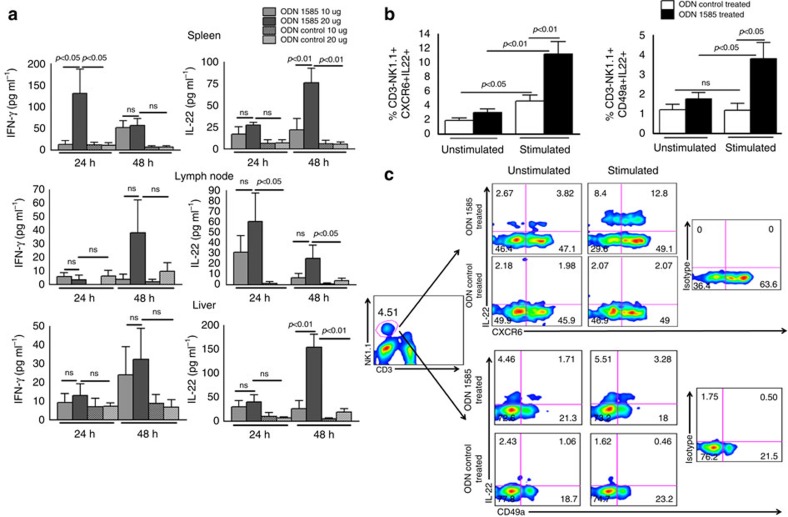
Immunization with a TLR9 agonist enhances the expansion of IL-22-producing NK1.1 cells. (**a**) The TLR9 agonist ODN 1585 enhanced IL-22 production by liver NK1.1 cells. Spleen, liver and lymph node cells from C57BL/6 mice were cultured with the TLR9 agonist ODN 1585 or ODN control for 24 and 48 h, and various cytokine levels were measured by ELISA. Bar graphs show the means±s.d. *P* values were generated by one-way analysis of variance (ANOVA). (**b**) Immunization of T1D mice with the same TLR9 agonist enhances the expansion of IL-22-producing CD3-NK1.1^+^ cells. C57BL/6 T1D mice were immunized intravenously with the TLR9 agonist ODN 1585 or treated with control ODN (20 μg per mouse). After 30 days, liver cells were isolated and restimulated with ODN 1585 or control ODN (20 μg ml^−1^). After 120 h, the expansion of CD3-NK1.1^+^CXCR6+IL-22+ and CD3-NK1.1^+^ CD49a+IL-22+ cellular populations was determined by flow cytometry. Bar graphs show the means±s.d. *P* values were generated by one-way analysis of variance (ANOVA). (**c**) A representative flow cytometry figure is shown. The data from five independent experiments are shown, and five mice were used per group.

Toll-like receptor 9 (TLR9) signalling can induce tolerance^24^, and NK1.1 cells express TLR9 (ref. 25). We asked whether using TLR9 agonists as adjuvants could enhance pancreatic islet allograft survival in liver parenchyma. We first determined IFN-γ and IL-22 production by cells obtained from liver and other lymphoid organs in the presence of either the TLR9 agonist CpG oligodeoxynucleotides (ODN 1585) or control oligodeoxynucleotides. C57BL/6 mouse spleen, lymph node and liver cells were cultured with varying concentrations of ODN 1585 or ODN control, and culture supernatants were collected at various time points. IFN-γ and IL-22 levels were measured in the culture supernatants by ELISA, as mentioned in the methods section. As shown in Fig. 6a, ODN 1585 significantly enhanced IFN-γ production by the spleen cells (*p*<0.05) compared with the ODN control. In contrast, we did not observe a statistically significant difference in IFN-γ production by liver or lymph node cells with ODN 1585 treatment. ODN 1585 significantly enhanced IL-22 production by spleen cells (*p*<0.01; ANOVA test), lymph node cells (*p*<0.05, ANOVA test) and liver cells (*p*<0.01, ANOVA test) compared with cells treated with the ODN control (Fig. 6a).

We next vaccinated the T1D mice with ODN 1585 or the ODN control. One month after vaccination, liver lymphocytes were isolated and restimulated with ODN 1585 or ODN control. As shown in Fig. 6b,c, ODN 1585 significantly expanded the IL-22-producing CD3-NK1.1^+^CXCR6+IL-22+ (4.61±0.85 versus 11.20±1.75; *p*<0.01, ANOVA test) and CD3-NK1.1^+^CD49a+IL-22+ (1.18±0.34 versus 3.80±0.81; *p*<0.05, ANOVA test) liver cells of ODN 1585-vaccinated mouse compared with ODN control-treated mouse liver cells, as determined by flow cytometry. Approximately 65% of the liver CD3-NK1.1^+^CXCR6+ cells were positive for NKG2A receptor (Supplementary Fig. 8), suggesting that majority of IL-22-producing CD3-NK1.1^+^CXCR6+ cells in the liver were hypo-responsive. As shown in Supplementary Fig. 9a, CD3-DX5-NK1.1^+^ or CD3-DX5+NK1.1^+^ cells from ODN 1585-vaccinated and restimulated with ODN 1585 are not cytotoxic. Islet viability was unaffected by CD3-DX5-NK1.1^+^ or CD3-DX5+NK1.1^+^ cells in the presence or absence of ODN 1585 or ODN control (Supplementary Fig. 9b).

**Figure 7:**
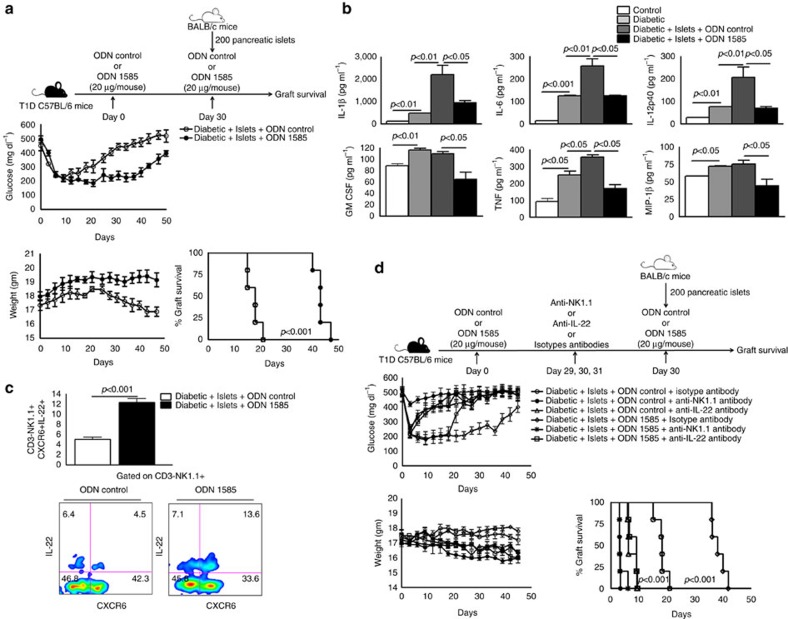
TLR9 agonist immunization before islet transplantation prolongs allograft survival. (**a**) Immunization of T1D mice with TLR9 agonist prolonged islet allograft survival. C57BL/6 T1D mice were immunized intravenously with the TLR9 agonist ODN 1585 or with ODN control (20 μg per mouse). Thirty days after immunization, pancreatic islets from BALB/c mice were transplanted as mentioned in Fig. 1. Blood glucose and body weight were measured every 72 h until 50 days. The *P* value for per cent graft survival was calculated using a log rank test. Kaplan–Meier survival curves of mice are shown. (**b**) Liver homogenates were prepared from transplanted mice after 20 days, and various cytokine levels were measured by ELISA. Bar graphs show the means±s.d. *P* values were generated by one-way analysis of variance (ANOVA). (**c**) After 20 days, liver lymphocytes were isolated. The expansion of NK1.1^+^CXCR6+IL-22+ cells in ODN 1585-vaccinated mice was determined by surface and intracellular staining. Bar graphs show the means±s.d. *P* values were generated by independent *t*-test. (**d**) IL-22 prolongs islet allograft survival in ODN 1585-vaccinated mice. C57BL/6 T1D mice were immunized intravenously with the TLR9 agonist ODN 1585 or with ODN control (20 μg per mouse). After 30 days, 200 pancreatic islets obtained from BALB/c mice (donor) were transplanted into the liver parenchyma of T1D C57BL/6 mice (recipients). Some of the islet allograft recipient mice were treated with anti-NK1.1 or anti-IL-22 or isotype control antibodies (0.5 mg per mouse 24 h before and 0 and 24 h after transplantation via tail vein injection). Blood glucose and body weight were measured every 72 h until 50 days. The *P* value for per cent graft survival was calculated using the log rank test. Kaplan–Meier survival curves of mice are shown. The data presented are representative of five independent experiments. Five mice were used per group.

Finally, we determined whether ODN 1585 vaccination enhanced pancreatic islet allograft survival in T1D mice. T1D mice were vaccinated with ODN 1585 or ODN control. After one month, these mice were transplanted with pancreatic islets obtained from BALB/c mice, as mentioned in Fig. 1 and the methods section. On the day of transplantation, ODN 1585-vaccinated-mice received ODN 1585, and ODN control-administered mice received ODN control along with the islets. The islet grafts survived for 47 days (*p*<0.001, log-rank test; Fig. 7a) in the ODN 1585-vaccinated mice and for 20 days in mice that received ODN control. In some of these experiments, liver lymphocytes were isolated from the above groups of mice 20 days after pancreatic islet transplantation, and real-time PCR was performed to determine the levels of cytokine expression. In ODN 1585-vaccinated mice, TGF-β (1.44±0.10 versus 5.64±1.37; *p*=0.03; ANOVA test), IL-22 (18.67±1.85 versus 31.23±4.10; *p*=0.04, ANOVA test) and IL-23 (1.537±0.28 versus 4.08±0.72; *p*=0.03, ANOVA test) expression was upregulated, and TNF (7.99±0.98 versus 1.82±0.35; *p*=0.004, ANOVA test; Supplementary Fig. 10) expression was downregulated compared with ODN control-treated mice. After 20 days of pancreatic islet transplantation, we also analysed chemokine and cytokine profiles in the liver homogenates of the above groups of mice using multiplex ELISA. As shown in Fig. 7b, various pro-inflammatory chemokine and cytokine levels were decreased in the liver supernatants of islet-transplanted ODN 1585-vaccinated mice compared with islet-transplanted ODN control-vaccinated mice. After 20 days of pancreatic islet transplantation, we found a significant expansion of CD3-NK1.1^+^CXCR6+IL-22+ cells in ODN 1585-vaccinated mice compared with ODN control-treated mice (Fig. 7c). We determined whether the increased islet allograft survival in the ODN 1585-vaccinated mice depends on IL-22 production. TID mice were vaccinated with the ODN 1585 or treated with ODN control. After 30 days, islets from BALB/c were transplanted into the liver parenchyma of the above mentioned T1D C57BL/6 mice, as described in the methods section. Some of the islet-transplanted T1D mice also received anti-NK1.1 or anti-IL-22 or isotype control antibodies one day before and one day after the transplantation via an intravenous injection. On the day of transplantation, mice received antibodies along with the islets. The islet allograft survived up to 40 days (*p*<0.001, log-rank test; Fig. 7d) in the ODN 1585-vaccinated mice that received isotype control antibodies. In contrast, in ODN 1585-vaccinated mice that received anti-NK1.1 and anti-IL-22 antibodies, islet allografts were rejected within 5 and 9 days, respectively (*p*<0.001, log-rank test; Fig. 7d).

## Discussion

In the current study, we found that liver NK1.1 cells induce pancreatic islet allograft tolerance in T1D mice. The tolerogenic effects of NK1.1 cells were mediated through the production of IL-22, which enhanced the survival of islets and increased the secretion of insulin. We also found that NKG2A expressed by liver NK1.1 cells is involved in the production of IL-22 and that inflammatory responses to the allograft were reduced. Furthermore we found that a CpG oligonucleotide-based adjuvant therapy in the T1D mice enhanced the expansion of IL-22-producing NK subsets in the liver and prolonged allograft survival. Our study identifies a novel role for liver NK1.1 cells and for IL-22 and CpG oligonucleotides in the induction of tolerance to islet allografts in the liver parenchyma.

The liver is known as an organ with an immunosuppressive environment, and ∼90% of clinical islet transplantations have been performed via the portal vein^26^,^27^,^28^. However, shortly after intraportal transplantation, many islets are lost because of an instantaneous blood-mediated inflammatory reaction (IBMIR), which is caused by the activation of the complement and coagulation cascades. Similarly, liver ischaemia contributes to the early failure of intraportal islet transplantation^28^. In the current study, we induced T1D in C57BL/6 mice and transplanted pancreatic islets obtained from BALB/c mice (allogeneic islets) into the liver parenchyma of T1D C57BL/6 mice to avoid IBMIR, thrombosis and ischaemia that result from the direct introduction of islets into intraportal blood. Our STZ-induced TID model differs with autoimmune diabetes models (autoimmune features occurs at late phase)^29^ in the development of T1D, but mimics T1D in terms of the loss of islets and deficiency of internal insulin. We found that allogeneic islets in the liver parenchyma of recipient T1D mice survived up to 15–20 days without any immunosuppressive treatment, as was previously reported. To determine the role of NK1.1 cells on islet allograft survival in liver parenchyma, we blocked NK1.1 cells by using an NK1.1 antibody and found that blocking NK1.1 cells at the time of the islet allograft transplantation induced graft rejection within 3–4 days. Our findings suggest an important role for liver NK1.1 cells in the induction of tolerance to islet allografts.

NK1.1 cells are a major immune cell population in the liver^30^, and there are conflicting reports regarding their role in the induction of allograft tolerance. NK1.1 cells can recognize transplanted tissue, acquire cytolytic effector functions, release a series of pro-inflammatory cytokines including IFN-γ and TNF and contribute to the rejection of solid organ allograft^31^,^32^,^33^. Similarly, liver NK1.1 cells destroy islet allografts after intraportal transplantation^34^. Islet allografts survive for longer-terms in CD1-deficient recipient mice, which do not possess NKT cells^18^. In contrast to these findings, studies have suggested that NK1.1 cells promote allograft survival by APCs and by secreting IL-10, thereby promoting the response of T-regulatory cells^17^,^35^. In the current study we found that depletion of NK1.1 cells during islet transplantation induced early allograft rejection, which suggests that liver NK1.1 cells induce tolerance to islet allografts. In Fig. 1, we show that treatment with anti-NK1.1 antibodies at the time of islet allograft transfer significantly enhanced the production of various inflammatory cytokines, but reduced IL-22 production. We found that there was a significant increase in the frequency of IL-22-producing CD3-DX5-NK1.1^+^ cells in the liver of islet allograft recipient mice within 3 days (Fig. 2a). Neutralization of IL-22 at the time of islet allograft transfer leads to graft rejection within 6 days, and administration of recombinant IL-22 prolonged the allograft survival. In addition, our data demonstrate that adoptive transfer of CD3-DX5-NK1.1^+^ cells after islet allograft transplantation prolongs islet allograft survival (Fig. 4a), and this prolonged survival was inhibited by anti-IL-22 antibody (Fig. 4b). Approximately 40% of adoptively transferred CD3-DX5-NK1.1^+^ cells produced IL-22 after seven days in the recipient T1D mouse liver (Supplementary Fig. 6b). Our findings demonstrate that IL-22-producing liver DX5-CD3-NK1.1^+^ cells play a critical role in islet allograft survival.

IL-22 is a member of the IL-10 cytokine family. IL-22-producing cells on mucosal surfaces are critical for enhancing epithelial resistance to injury, stimulating the production of antimicrobial proteins and protecting against bacteria that cause intestinal disease and pneumonia^36^,^37^. IL-22-deficient mice are highly susceptible to concanavalin-A-mediated liver inflammation^36^, and overexpression of IL-22 via gene delivery attenuates experimental concanavalin-A- and FAS-mediated liver damage, suggesting a protective role during acute liver inflammation^38^. IL-22 is important for the maintenance of homoeostasis, and it protects major organs in transplant recipients from graft versus host disease (GVHD)^39^. Deficiency in IL-22 contributes to a chronic inflammatory disease through the increased expression of chemokines, such as CXCL1, CXCL2 and CCR5, and inflammatory cytokines, such as IL-17A, IL-26, IFN-γ, IL-24, and IL-1β (ref. 40). Preclinical studies demonstrate that recombinant IL-22 has broad therapeutic potential against epithelial cell injury^41^. IL-22 is produced by activated T, NK, NKT and γδ T cells^42^. We found that the numbers of IL-22-producing T cells were similar in control and islet allograft-transplanted mice and that DX5-CD3-NK1.1^+^ cells are a major source of IL-22 (Fig. 2a). Liver γδ T cells in allograft recipient mice also produced marginal amounts of IL-22.

The mechanism by which IL-22 prolongs islet allograft survival in the liver parenchyma is not known. During transplantation, islets are maintained under hypoxic conditions until they are re-vascularized by the host fluidic system. Hypoxic conditions induce oxidative and ER stress in islets, which can lead to the destruction of the islets^43^,^44^. IL-22 is a powerful endogenous paracrine suppressor of oxidative and ER stress in pancreatic islets^21^,^45^. We found that recombinant IL-22 enhanced the survival of pancreatic islets and the secretion of insulin by islets. Culturing pancreatic islets with liver lymphocytes enhanced insulin production, and this was inhibited by anti-IL-22 antibodies and enhanced by the addition of recombinant IL-22. We also found that the addition of CD3-DX5-NK1.1^+^ cells to NK1.1 cell-depleted liver cells and islets enhanced IL-22 and insulin secretion, suggesting IL-22-producing liver CD3-DX5-NK1.1^+^ cells regulate the insulin secretion by islet allografts. Pancreatic β-cells express IL-22R1 (Supplementary Fig. 5), and IL-22 therapy restores glucose control by attenuating defects in β-cell insulin biosynthesis and secretion^21^,^45^,^46^. Recently, Park *et al*. found that IL-22 inhibits hepatic gluconeogenesis and reduces blood glucose levels on short-term exposure (2 h). In the current study, we found that IL-22 enhances allograft survival in the transplanted mouse liver parenchyma. We also found that IL-22 enhances the expression of insulin-producing genes and insulin production by islets 72 h after stimulation. The difference between the study conducted by Park *et al*. and our study may be that Park *et al*. found acute effects of rIL-22, while the current study showed chronic effects of rIL-22. Our current findings demonstrate a novel role for IL-22 produced by liver NK1.1 cells during islet allograft transplantation in the liver parenchyma, but the exact mechanisms of IL-22 function are not completely known. Further studies are needed to elucidate the mechanisms of IL-22 function in islet allograft survival.

We found that culturing pancreatic islets with recombinant IL-22 enhanced the expression of the regeneration genes (*Regs*) *Reg2*, *Reg3a* and *Reg3*, which are known to induce β-cell regeneration and reverse T1D in mice^47^. Our findings are in accordance with previous findings showing that IL-22 enhanced the expression of *Reg2* and *Reg1* by β-cells. In addition, we found that IL-22 enhanced *Pdx1* and *IRE1* expression by pancreatic islets (Fig. 3e). *Pdx1* is also known as insulin promoter factor 1 and is a transcription factor necessary for pancreatic development, β-cell maturation and insulin secretion^48^,^49^. *IRE1* is an ER-resident transmembrane protein kinase that regulates insulin secretion from the ER (ref. 50). Our findings demonstrate that IL-22 produced by liver NK1.1 cells enhances the expression of *Reg* genes, the *Pdx1* transcription factor and a transmembrane protein kinase *IRE1* by pancreatic islets to enhance their survival and insulin secretion in the liver parenchyma.

Depletion of NK1.1 cells in islet allograft recipient mice at the time of transplantation induced a robust allogeneic immune response in the liver within the first 3–4 days by upregulating pro-inflammatory cytokine expression (Fig. 1). Our findings demonstrate that in addition to producing IL-22, which enhances the survival and insulin secretion by pancreatic islets, liver NK1.1 cells can prevent early inflammatory responses to allografts. The liver environment regulates NK cell receptor expression and contains a significant population of functionally hypo-responsive NK cells, suggesting that an NK cell activating or inhibitory phenotype plays an important role in regulating immune responses. Among the various inhibitory receptors tested, only NKG2A expression by NK cells was upregulated at early time points after transplantation (3 days after transplantation). In contrast, at a later time point (15 days after transplantation), when allograft rejection is occurring, we found increased expression of the activating receptor NKG2D on liver NK cells. NK cells reduce neutrophil recruitment and inhibit the inflammatory response through NKG2A receptors, and blocking NKG2A receptors induces hyper-activation of immune cells and inflammatory cytokine production^22^. In the current study, we found that anti-NKG2A antibody significantly enhanced inflammatory cytokine production by recipient mouse liver lymphocytes that were cultured with pancreatic islets. Surprisingly, anti-NKG2A antibodies inhibited IL-22 and insulin production. NKG2A expression plays an important role in liver dendritic cells, which induce the expansion of T-regulatory cells^51^. We found that the depletion of NK1.1 cells had no effect on Foxp3 expression but significantly decreased the expression of IL-10 by recipient mouse liver cells (Fig. 1), suggesting that CD3-DX5-NK1.1^+^ cells may enhance IL-10 production by APCs to inhibit excess inflammation.

Intravenous administration of the TLR9 agonist ODN1585 in T1D mice one month before pancreatic islet transfer and at the time of islet transplantation significantly enhanced graft survival compared with treatment with control ODN. TLR9 is expressed by different cell types, including B cells, macrophages, DC and NK cells^25^,^52^, and the tolerogenic effect depends on the location of these cell populations and the route of administration of the TLR9 agonists^53^,^54^. Subcutaneous administration of CpG ODN along with the antigen results in antigen-specific T cell activation in lymph nodes^54^. CpG-containing ODN activates peripheral NK cells via TLR9 and enhances NK cell cytokine secretion and lytic activity^25^. In contrast, we found that ODN 1585-cultured liver NK1.1 cells produce IL-22, are not cytolytic and do not affect the viability of allogeneic islets. The systemic application of CpG-containing ODN leads to the selective IDO-dependent suppression of T cell expansion and cytolytic activity^24^. A series of recent studies demonstrated that administration of TLR9 agonist (CpG-rich oligonucleotides) in various experimental settings leads to the activation of immunosuppressive pathways^24^,^53^,^55^,^56^. In other studies, TLR9 stimulation promoted pDC-mediated generation of CD4+CD25+T-regulatory cells^55^, TGF-β-dependent immune suppressive tryptophan catabolism and IDO expression to maintain tolerance^24^,^55^,^56^. In the current study, we found that ODN 1585 administration before islet allograft transplantation in T1D mice induced the expansion of IL-22-producing liver NK cells. Our study demonstrates that the activation of hypo-responsive liver NK1.1 cells via TLR9 stimulation can lead to the expansion of IL-22-producing NK1.1 cells, which can enhance islet allograft survival in the liver.

In summary, our study demonstrates that liver IL-22-producing NK1.1 cells are essential for the maintenance of islet allograft tolerance in the liver. Our study provides evidence that IL-22 contributes to the survival of islet allografts in the liver and enhances insulin production. We also found that the TLR9 agonist ODN 1585 can be used to prolong pancreatic islet allograft survival in the liver.

## Methods

### Animals

Female of 6- to 8-week-old C57BL/6 and 11- to 12-week-old female BALB/c mice were purchased from the Jackson Laboratories. The Institutional Animal Care and Use Committee of the University of Texas Health Science Center at Tyler approved the studies. All animal procedures involving the care and use of mice were in accordance with the guidelines of NIH/OLAW (Office of Laboratory Animal Welfare).

### Islet isolation and transplantation

Islets were isolated from the pancreas of BALB/c or C57BL/6 mice by collagenase V and VI digestion (Sigma-Aldrich) and purified by using a density gradient method^57^. C57BL/6 recipient mice received one intraperitoneal injection of 180 mg kg^−1^ body weight of streptozotocin (Sigma-Aldrich) to induce type 1 diabetes^58^. After 7–10 days of the induction of type 1 diabetes, each mouse received ∼200 islets (from BALB/c mice), which were grafted into the liver parenchyma.

### Graft survival analysis

In control and islet-transplanted mice, blood glucose levels were measured every 72 h until the mice were killed. Blood samples were collected by submandibular bleeding using a strip glucometer (Elite, Bayer). Graft survival was calculated as the number of days before diabetes recurrence. The day of diabetes recurrence was defined as the first of two consecutive days of random blood glucose >300 mg dl^−1^.

### Islet viability and function

The viability of islets was analysed under each condition by fluorescein diacetate/propidium iodide staining (FDA/PI, Sigma-Aldrich), which was performed by two independent investigators. The ratio of green to red cells determined the percentage of viable cells^59^. Images were obtained using an Olympus IX51 microscope. The results are expressed as the percentage of viable cells. To determine islet function, 100 pancreatic islets were cultured in RPMI 1640 media with 10% FBS and 5.5–10 mM glucose (Sigma-Aldrich). Each incubation step was performed for 120 min at 37 °C in a humidified 5% CO_2_ atmosphere. In some *in vitro* experiments, islets were cultured in the presence of recombinant cytokines for 24 and 48 h at 37 °C in a humidified 5% CO_2_ atmosphere. Supernatants were collected and stored at −80 °C. Insulin levels in culture supernatants were measured using a mouse insulin enzyme-linked immunosorbent assay (ELISA) kit (Mercodia). The results are expressed as ng per ml insulin released per 100 islets.

### Antibody and cytokine treatment

In some experiments, pancreatic islet allograft recipient mice also received anti-NK1.1 or isotype control antibodies (BioXcell, clone PK136, 0.5 mg per mouse) 24 h before and 0 and 24 h after transplantation via a tail vein injection^60^. In other experiments, some of the pancreatic islet allograft recipient mice also received anti-IL-22 (eBioscience, clone JES6-1A12, 0.3 mg per mouse), anti-NKG2A (Bio-Rad, clone 20D5, 0.3 mg per mouse)^22^,^23^ or isotype control antibodies (Rat IgG2a K isotype control antibody, eBioscience, Clone: eBR2a, 0.3 mg per mouse) 24 h before and 0 and 24 h after transplantation via a tail vein injection.

Recombinant IL-22 (BioLegend) (100 ng g^−1^ body weight) or normal saline was administered to some allograft recipient mice via an intravenous injection. For *in vitro* experiments, islets were cultured in the presence of recombinant IL-22 or IFN-γ (BioLegend) at a concentration of 10–20 ng ml^−1^ for 24 and 48 h.

### Liver cell isolation

The control group and islet transplanted T1D mice were killed, and perfused with 10 ml of solution (0.5 M EDTA in 1 × Hanks Balanced Salt Solution without Ca^2+^ and Mg^2+^). The perfused liver was removed and mechanically disintegrated in a petri dish containing 1 × PBS. The single cell suspensions of liver cells were filtered through a 70 μm mesh filter, and the resulting cell suspension was centrifuged at 1500, r.p.m. for 5 min.

### Isolation of liver lymphocytes

In some experiments, control group mice and islet-transplanted T1D mice were killed, and liver single cell suspensions were prepared as described in the liver cell isolation section. Liver lymphocytes were isolated with the differential density gradient method using a Percoll (Sigma-Aldrich) density gradient^61^,^62^.

### Sorting of NK1.1^+^ cells

Liver CD3-DX5-NK1.1^+^ or CD3-DX5+NK1.1^+^ cells or splenic CD3-DX5+NK1.1^+^ cells were sorted using a FACSAria cytometer (BD Biosciences). From whole liver cells, the purity of the flow cytometry-sorted cell population and the above immunomagnetically isolated cell population was more than 99%. Flow-sorted liver CD3-DX5-NK1.1^+^ or CD3-DX5+NK1.1^+^ cells or splenic CD3-DX5+NK1.1^+^ cells from CD45.2 C57BL/6 mice were adoptively transferred (10^5^) through tail vein injection to CD45.1 T1D C57BL/6 mice. For some experiments, mouse liver and splenic NK1.1 (DX5+ and DX5-) cells were isolated using an immunomagnetic negative selection method (Miltenyi Biotec, Inc., San Diego, CA, USA).

### In vitro allogeneic co-culture

Whole liver or NK1.1 cell-depleted liver cells (1 × 10^5^) from T1D C57BL/6 mice were cultured with 10 pancreatic islets from BALB/c mice in 12-well plates with RPMI 1640 (Gibco) containing 10% heat-inactivated fetal bovine serum (Gibco) at 37 °C in a humidified 5% CO_2_ atmosphere for three days^63^,^64^. To some wells, anti-IL-22 (eBiosciences, clone: IL22JOP), anti-NKG2A (eBiosciences, clone: 20d5) antibody or isotype control antibody (eBiosciences, clone: eBR2a) (1.5 μg ml^−1^) were added on day 0. After three days, culture supernatants and cell lysates were collected and stored at −80 °C for further analysis.

### Preparation of liver homogenates for cytokine measurements

Islet-recipient mice were killed, and their livers were rapidly excised, rinsed of blood and homogenized in homogenization buffer (PBS containing 0.05% sodium azide, 0.5% Triton X-100 and a protease inhibitor cocktail, pH 7.2, 4 °C). Homogenates were centrifuged at 18,000 r.p.m. for 10 min, and the supernatants were used for various experiments. Cytokine levels in the liver homogenate were measured by multiplex ELISA or using individual ELISA kits.

### Multiplex ELISA

In the culture supernatants and liver homogenates, the following 27 cytokines and chemokines were measured using a multiplex ELISA kit (M60009RDPD, Bio-Rad). The cytokines and chemokines analysed were IL-1β, IL-1ra, IL-2, IL-4, IL-5, IL-6, IL-7, IL-8, IL-9, IL-10, IL-12 (p70), IL-13, IL-15, IL-17, basic FGF, Eotaxin, G-CSF, GM-CSF, IFN-γ, IP-10, MCP-1 (MCAF), MIP-1α, MIP-1β, PDGF-BB, RANTES, TNF and VEGF. In some experiments, IL-22, IL-1α, IL-12 (p70), MIP-1α, IL-17, IFN-γ, IL-1β (88-7422-88, 88-5019-88, 88-7121-88, 88-56013-88, 88-7371-88, 88-7314-88 and 88-7013-88 from eBiosciences), KC, MIP-1β (DY453-05 and DY451 from R&D) and insulin (10124701, Mercodia) levels were measured using individual ELISA kits.

### Real-time PCR for quantification

RNA was isolated from lymphoid organs and from the liver lobes that contained grafts (harvested at various time points after transplantation) using TRIzol (Invitrogen) according to the manufacturer's instructions. complementary DNA (cDNA) was generated from 0.5 mg RNA and random hexamer primers using the Maxima First Strand cDNA Synthesis Kit for RT-qPCR (BIO-RAD), according to manufacturer's instructions, and real time PCR was performed as previously reported^65^. Gene expression of the immune system-related genes IL-1β, TNF, IFN-γ, IL-10, IL-6, FoxP3, TGF-β, IL-18, IL-23 and IL-22 was quantified in the lymphoid organs, and the cell regeneration genes *Reg1a, Reg2, Reg3a* and *Reg3g* and insulin secretion-regulating genes *pdx1, Foxa2, IRE1 and TXNIP* were measured in cytokine-treated islets using the Syber green master mix (Qiagen) and gene-specific primers (Sigma-Aldrich) (Supplementary Table 1) on an ABI Prism 7600. All gene expression levels were normalized to GAPDH and/or β-actin internal controls, and fold changes were calculated using the 2^−ΔΔ*CT*^ method.

### siRNA transfection

Liver cells were transfected with the NKG2A or Qa-1 (Santa Cruz Biotechnology) or control siRNA (Santa Cruz Biotechnology). In some experiments, freshly sorted CD3-DX5-NK1.1^+^ cells were transfected with siRNA for IL-22. The efficiency of siRNA knockdown was measured by real-time PCR. Briefly, 10^6^ liver or CD3-DX5-NK1.1^+^ cells were resuspended in 500 μl of transfection medium and were transfected with siRNA (6 pmoles). After 6 h, an additional 250 μl of 2 × RPMI complete medium was added, and cells were cultured overnight in a 24-well plate. The next day, liver or CD3-DX5-NK1.1^+^ cells were washed and used for experiments.

### Antibodies and other reagents

For flow cytometry, PE–Cy5 anti-NK1.1 (5 μl per 100 μl of sample, clone: PK136), FITC anti-CD3 (5 μl per 100 μl of sample, clone: 17A2), APC anti-CD3 (5 μl per 100 μl of sample, clone: 17A2), FITC anti-DX5 (5 μl per 100 μl of sample, clone: DX5), FITC anti-CD4 (5 μl per 100 μl of sample, clone: RM4-5), APC anti-CD8 (5 μl per 100 μl of sample, clone: SK1), FITC anti-γδ TCR (5 μl per 100 μl of sample, clone: GL3), APC anti-Ly49A (5 μl per 100 μl of sample, clone: A1/Ly49A), PE anti-NKG2A (5 μl per 100 μl of sample, clone: 16A11), PE anti-NKG2D (5 μl per 100 μl of sample, clone: 1D11), APC anti-KLRG1 (5 μl per 100 μl of sample, clone: 2F1/KLRG1), PE anti-IL-22 (5 μl per 100 μl of sample, clone: Poly5164), APC anti-IL-22 (5 μl per 100 μl of sample, clone: Poly5164), PE anti-IFN-γ (5 μl per 100 μl of sample, clone: XMG1.2), APC anti-CD49a (5 μl per 100 μl of sample, clone: HMα1), APC anti-mouse CD326 (Ep-CAM) (5 μl per 100 μl of sample, clone: G8.8) and APC anti-CXCR6 (5 μl per 100 μl of sample, clone: K041E5) (all from BioLegend), Anti- mouse IL-22R alpha 1 (2.5 μg per 10^6^ cells, clone: 496514), Rat IgG2A Isotype Control (2.5 μg 10^−6^ cells, clone: 54447) and Anti mouse Proinsulin (2.5 μg per 10^6^ cells, clone: 253627) (from R&D systems) were used.

### Flow cytometry

For surface staining, 10^6^ cells were resuspended in 100 μl of staining buffer (PBS containing 2% heat-inactivated fetal bovine serum (FBS)) and Abs. Cells were then incubated at 4 °C for 30 min, washed twice and fixed in 1% paraformaldehyde before acquisition on a FACSCalibur (BD Biosciences). In some experiments, intracellular staining was performed according to the manufacturer's instructions^65^.

### Immunofluorescence

Frozen sections of liver and pancreas (5 μm) were fixed in cold acetone for 10 min. After fixation, an FcR blocking step was performed at room temperature for 30 min. For insulin staining in the transplanted liver, monoclonal mouse primary anti-insulin antibody (Abcam) was used. In other experiments, pro-insulin and anti-IL-22R1 were used in pancreas sections stained with proinsulin (R&D systems) and IL-22R alpha 1 antibodies, respectively. Subsequently, the slides were washed thoroughly with 1 × PBS. Then, cells were stained with their respective secondary antibodies (goat anti-hamster IgG-Alexa 568, goat anti-rabbit-Alexa 568 and donkey anti-rat- Alexa 488). The cells were washed with PBS and mounted with Prolong Gold anti-fading reagent with DAPI (Life Technologies, USA). The slides were examined using a fluorescence microscope (Olympus BX 50). Immunostaining of paraffin sections was performed according to the manufacturer's instructions using antibodies against insulin (Abcam, USA) on paraffin-fixed thin sections^66^.

### Statistical analysis

Prism 4.03 software (GraphPad Software; San Diego, CA) was used for statistical analyses. *p* Values which were <0.05 were considered statistically significant. The results are shown as the means±SE. Comparisons between groups were performed by paired, unpaired *t*-test and one-way ANOVA, as appropriate. Islet allograft survival was determined by Kaplan–Meier survival curves, and the comparisons between groups were made with log-rank analyses.

### Data availability

The authors declare that the data supporting the findings of this study are available within the article and its Supplementary Information Files or from the corresponding authors on request.

## Additional information

**How to cite this article:** Tripathi, D. *et al*. A TLR9 agonist promotes IL-22-dependent pancreatic islet allograft survival in type 1 diabetic mice. *Nat. Commun.* **7,** 13896 doi: 10.1038/ncomms13896 (2016).

**Publisher's note:** Springer Nature remains neutral with regard to jurisdictional claims in published maps and institutional affiliations.

## Supplementary Material

Supplementary Information Supplementary Figures and Supplementary Table
