# Supplementary material for: A TLR9 agonist promotes IL-22-dependent pancreatic islet allograft survival in type 1 diabetic mice
Source: Nat Commun. 2016 Dec 16;7:13896. doi: 10.1038/ncomms13896 (PMC5171644; doi:10.1038/ncomms13896)
Supplement: Supplementary Information — Supplementary Figures and Supplementary Table [file ncomms13896-s1.pdf]

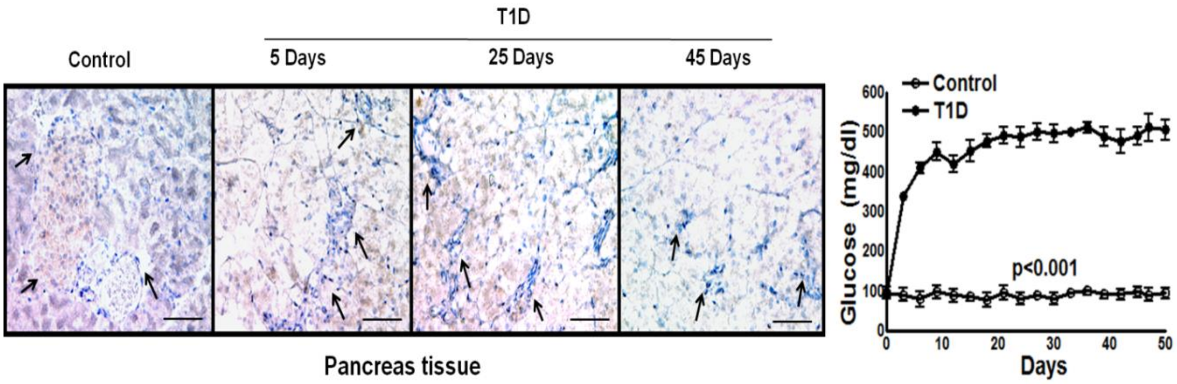

Supplementary Figure 1. Immunohistochemistry of control and T1D mouse pancreas. Immunohistochemical analyses of pancreas sections from control and T1D mice were performed on days 5, 25 and 45 after T1D induction. Photographs are representative of staining patterns. Magnification 20X; scale bar = 100 μm (Right). Blood glucose concentrations were measured every 72 hours until 50 days. Bar graphs show the means ± s.d. P values were generated by independent t-test. Five mice were used per group.

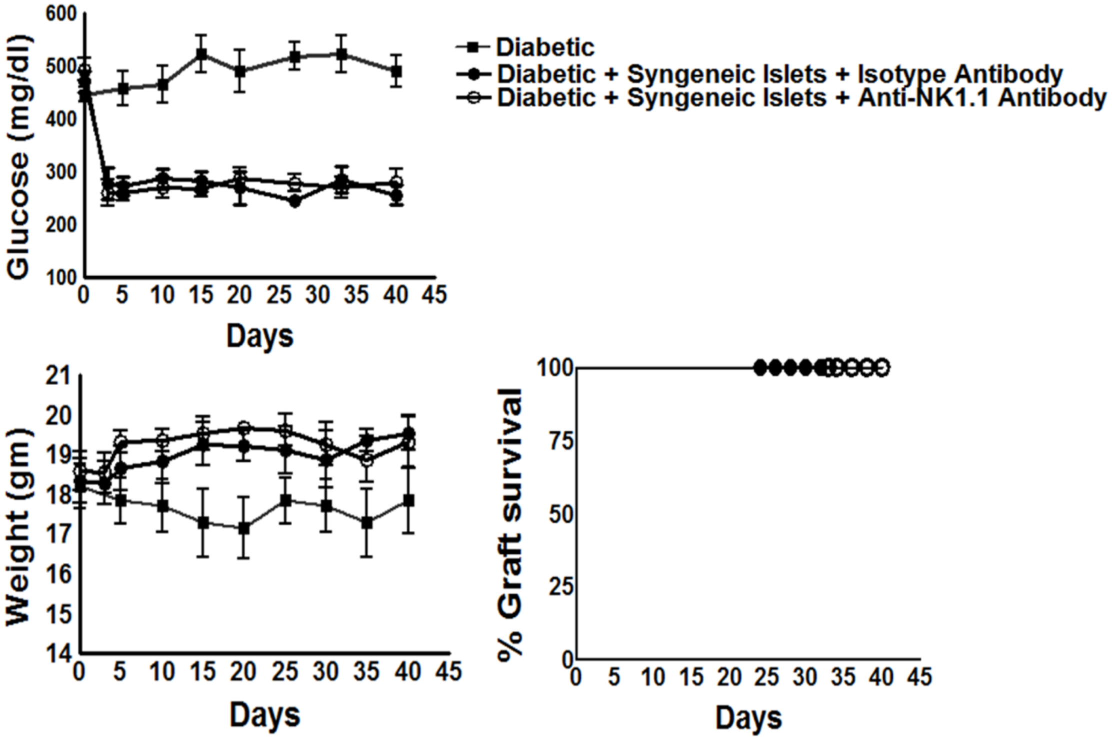

Supplementary Figure 2. Syngeneic pancreatic islet graft survival in T1D mice. (a) Approximately 200 pancreatic islets obtained from C57BL/6 mice (donor) were transplanted into the liver parenchyma of T1D C57BL/6 mice (recipients). Some of the islet allograft recipient mice were treated with anti-NK1.1 or isotype control antibodies (0.5 mg/mouse 24 hours before and 0 and 24 hours after transplantation via tail vein injection). Blood glucose and body weight were measured every 72 hours until 24 days. Blood glucose levels above 300 mg/dl were considered as failed glucose control. The P value for percent graft survival was calculated using the log rank test. Kaplan–Meier survival curves of mice are shown. The data presented are representative of two independent experiments, and five mice per group were used in each independent experiment.

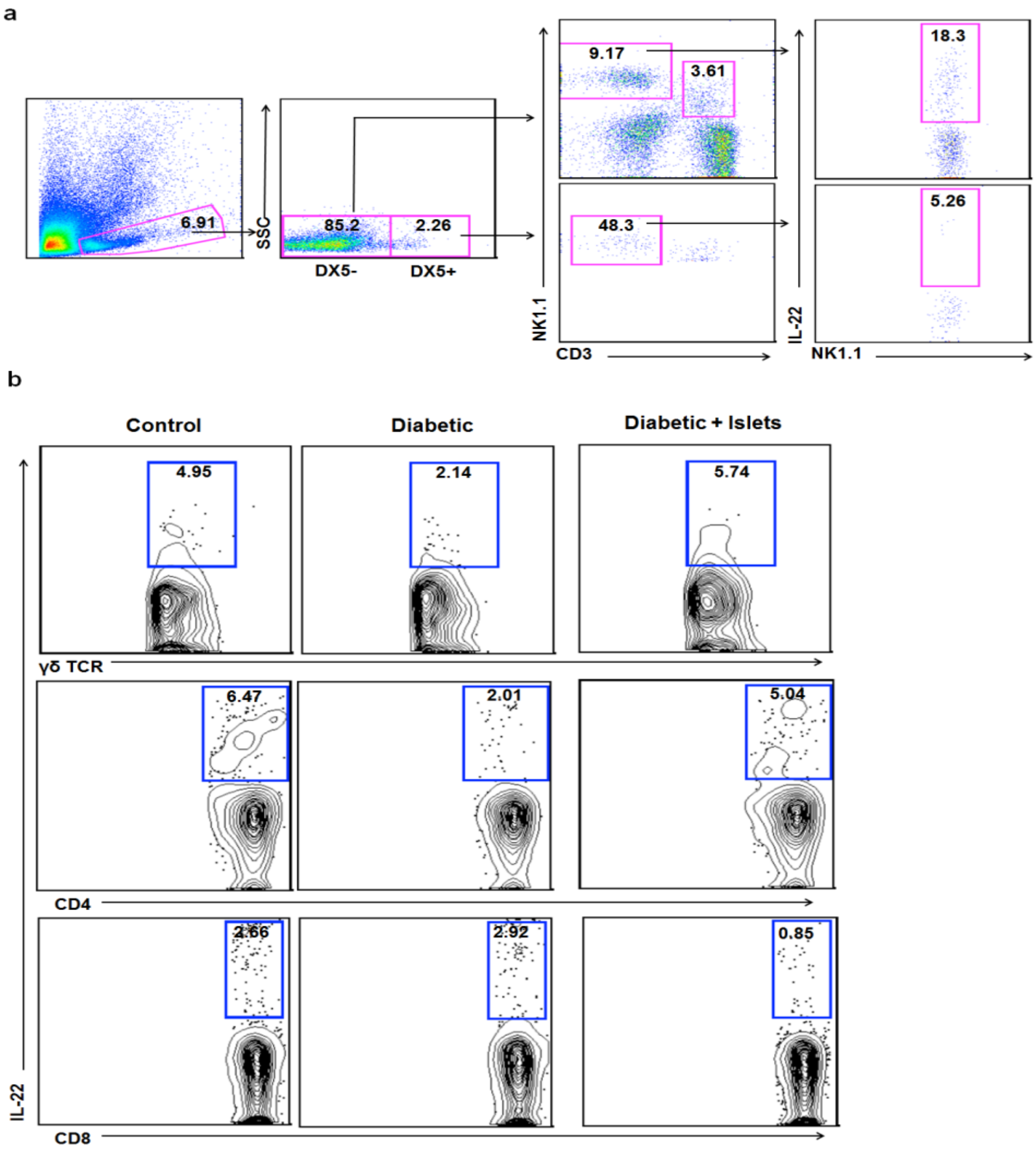

Supplementary Figure 3. IL-22 production by liver cells. Pancreatic islets from BALB/c mice were transplanted into the liver parenchyma of T1D C57BL/6 mice (recipients) as mentioned in Fig. 1. Three days after transplantation, the percentages of IL-22+ cells in the recipient liver were determined by flow cytometry. **(a)** A representative flow cytometry plot of gating strategies for DX5- and DX5+ IL-22-producing cells is shown. **(b)** A representative flow cytometry plot for

IL-22-producing γδ TCR+, CD4+ and CD8+ T cells is shown. The data shown in the figure are representative of five independent experiments. Five mice per group were used.

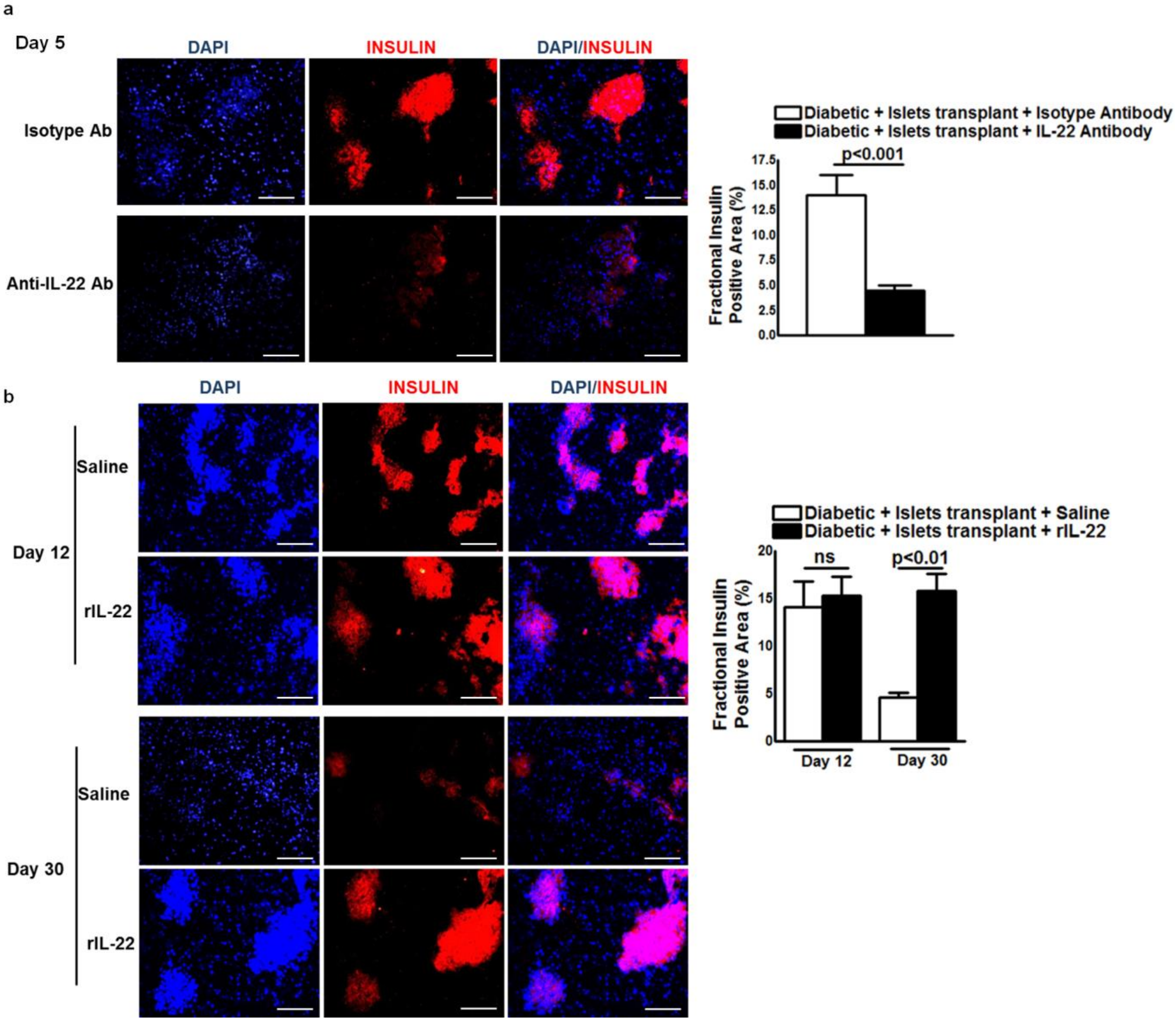

Supplementary Figure 4. IL-22 regulates insulin production by transplanted islet allograft. (a) Anti-IL-22 antibody treatment inhibits insulin production by pancreatic islets. Pancreatic islets from BALB/c mice were transplanted into the liver parenchyma of T1D C57BL/6 mice (recipients) as mentioned in Fig. 1. Some of the islet allograft recipient mice were treated with anti-IL-22 or isotype control antibodies (0.3 mg per mouse 24 hours before, 0 and 24 hours after transplantation through tail vein injection). Five days after islet allograft transplantation, recipient livers were stained for insulin (red) and DAPI (blue). Bar graphs show the means ± s.d. P values were generated by independent t-test **(b) Recombinant IL-22 treatment prolongs**

insulin secretion from islet allografts. Pancreatic islets from BALB/c mice were transplanted into the liver parenchyma of CD45.1 T1D C57BL/6 mice (recipients) as mentioned in Fig. 1. After 12 days, mice were treated with recombinant IL-22 (100 ng/kg of body weight) twice weekly for 50 days. On the 12th and 30th day, islet allograft-containing recipient livers were stained for insulin (red) and DAPI (blue). Photographs are representative of staining patterns. Magnification 20X; scale bar = 100 μm (Right). Bar graphs show the means ± s.d. P values were generated by independent t-test. The data shown are representative of three independent experiments.

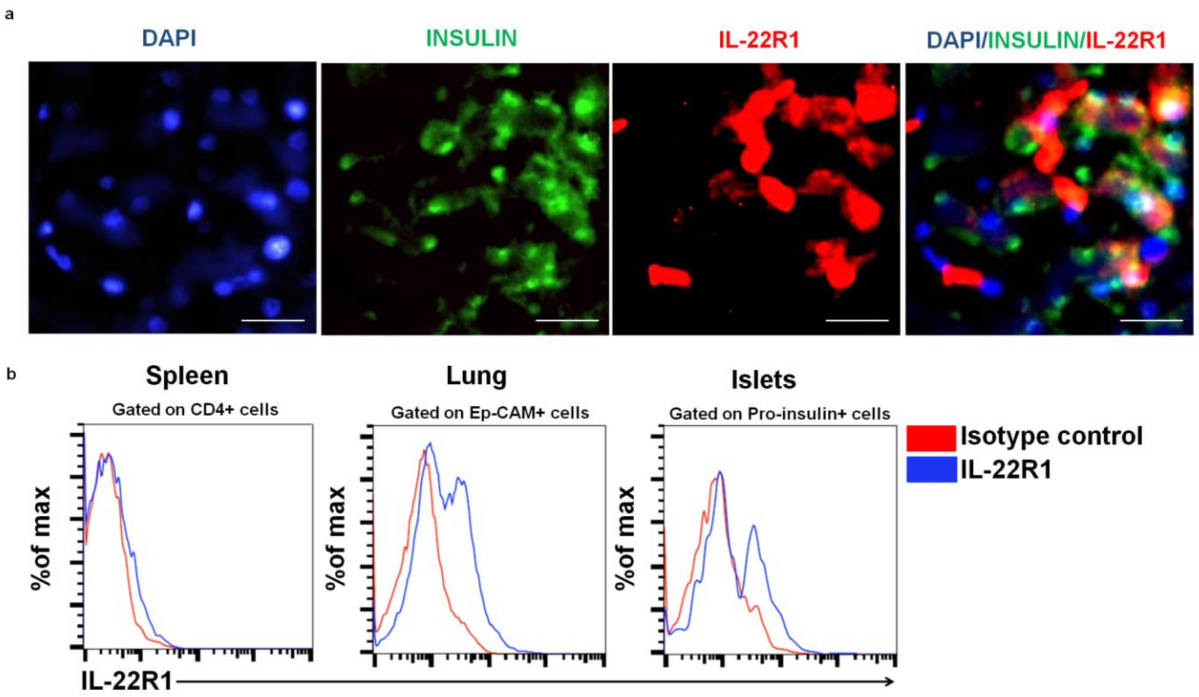

Supplementary Figure 5. Expression of IL-22R1 by insulin-producing pancreatic islets. (a) C57BL/6 mouse pancreas sections (5 μm) were stained for insulin (green), IL-22R1 (red) and DAPI (blue) by immunofluorescence microscopy. Photographs are representative of staining patterns. Magnification 40X; scale bar = 50 μm (Right). (b) Wild type C57BL/6 mouse spleen and lung single cell suspensions were prepared, and pancreatic islets were digested. Flow cytometry was performed on splenic CD4+ (negative control), lung Ep-CAM+ cells (lung epithelial cells) (positive control) and insulin-producing pancreatic islets (β-cells) to determine IL-22R1 expression using anti-IL-22R1 antibody. A representative flow cytometry diagram is shown. All data shown in the figure are representative of three independent experiments.

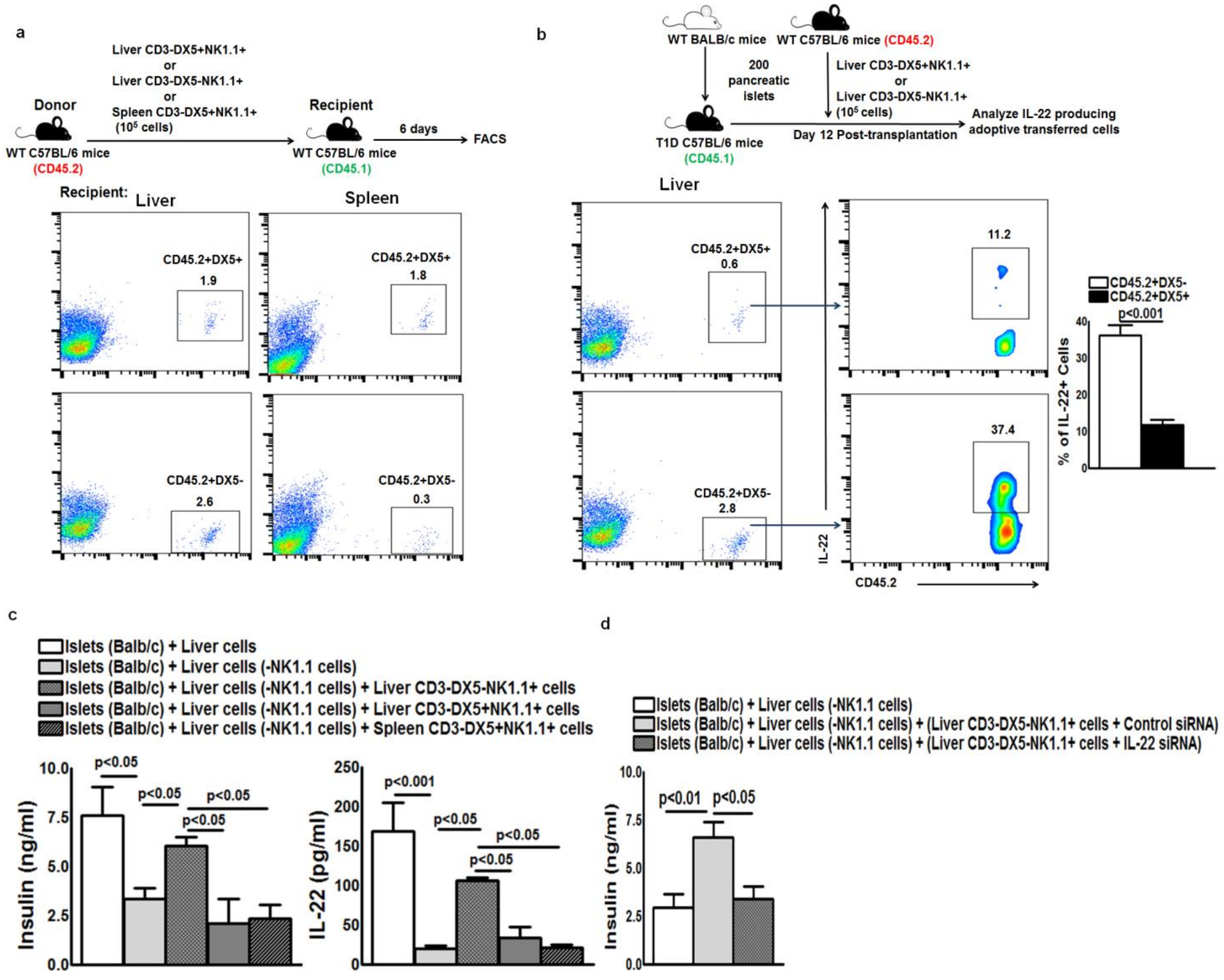

Supplementary Figure 6. IL-22-producing liver resident CD3-DX5-NK1.1+ cells prolong islet allograft survival. (a) Liver CD3-DX5-NK1.1+ or CD3-DX5+NK1.1+ or splenic CD3-DX5+NK1.1+ cells were sorted from control CD45.2 mice and given to CD45.1 TID C57BL/6 mice via tail vein injection. Six days after adoptive transfer, the percentages of DX5+CD45.2+ and DX5-CD45.2+ cells in liver and spleen are shown. **(b)** Pancreatic islets from BALB/c mice were transplanted into the liver parenchyma of CD45.1 T1D C57BL/6 mice (recipients) as mentioned in Fig. 1. Twelve days after transplantation, $1{\times}10^5$ liver CD3-DX5-NK1.1+ or CD3-DX5+NK1.1+ cells from control CD45.2 C57BL/6 mice were adoptively transferred via tail vein injection into CD45.1 T1D C57BL/6 mice (recipients) as mentioned in Fig. 1. Seven days after

adoptive transfer, IL-22-producing donor cells (CD45.2) were determined. Bar graphs show the means ± s.d. P values were generated by independent t-test. All data shown are representative of five independent experiments, and five mice were used per group **(c)** Whole liver cells or NK1.1 cell-depleted liver (NKDL) cells from T1D C57BL/6 mice were cultured with BALB/c mouse pancreatic islets at a ratio of 10,000:1 (10,000 liver cells and one islet), and some of the NKDL cells were cultured with 10^3 liver CD3-DX5-NK1.1+ or CD3-DX5+NK1.1+ or splenic CD3-DX5+NK1.1+ cells of control C57BL/6 mice. After 72 hours, insulin and IL-22 levels were measured by ELISA. Bar graphs show the means ± s.d. P values were generated by one-way analysis of variance (ANOVA). **(d)** The experiment was performed in the same manner as panel "c" except that some of the CD3-DX5-NK1.1+ cells were treated with IL-22 siRNA or control siRNA before culturing with NKDL cells and pancreatic islets. After 72 hours, insulin levels were measured by ELISA. Bar graphs show the means ± s.d. P values were generated by one-way analysis of variance (ANOVA). All data shown are representative of three independent experiments.

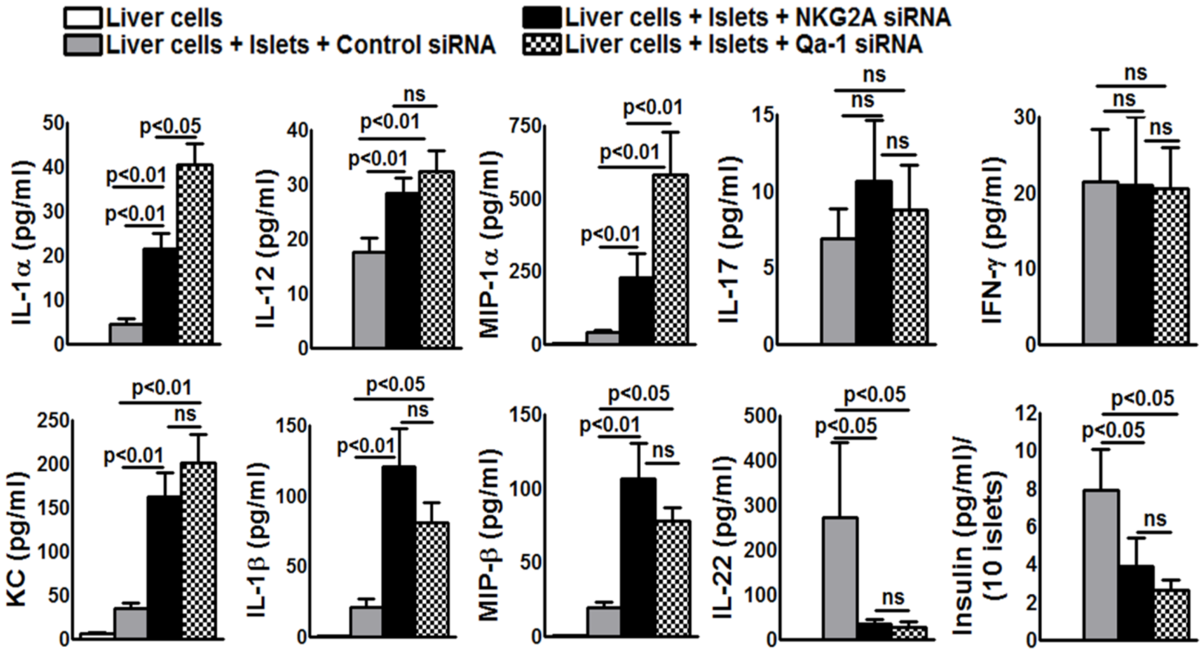

Supplementary Figure 7. NKG2A and its ligand Qa-1 inhibit the production of pro-inflammatory cytokines in response to islet allografts. (a) Liver cells from T1D mice were isolated and transfected with siRNA for NKG2A or Qa-1 or scrambled siRNA (control siRNA) and cultured with BALB/c mouse pancreatic islets at a ratio of 10,000:1 (1×10^5:10). After 72 hours, various cytokine levels were measured by ELISA. Bar graphs show the means ± s.d. P values were generated by one-way analysis of variance (ANOVA). The data shown are representative of three independent experiments.

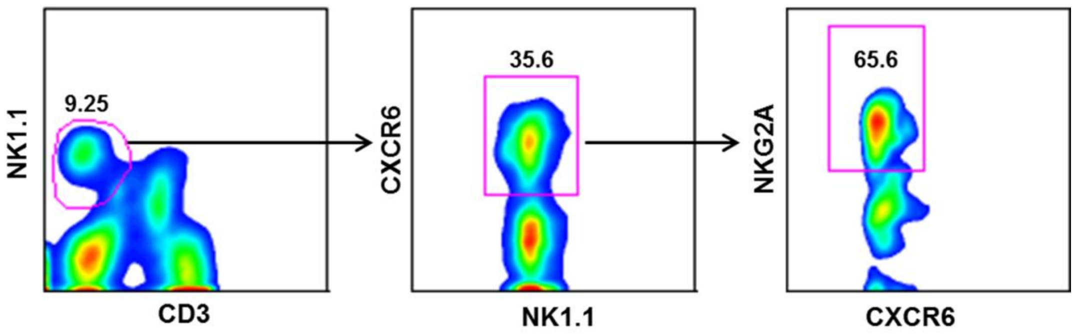

Supplementary Figure 8. Expression of NKG2A receptor by liver CD3-NK1.1+CXCR6+ cells. Liver lymphocytes were isolated from control C57BL/6 mice, and percentages of NKG2A+ cells in CD3-NK1.1+CXCR6+ cells were determined by flow cytometry. A representative flow cytometry plot is shown. All the data shown in the figure are representative of three independent experiments.

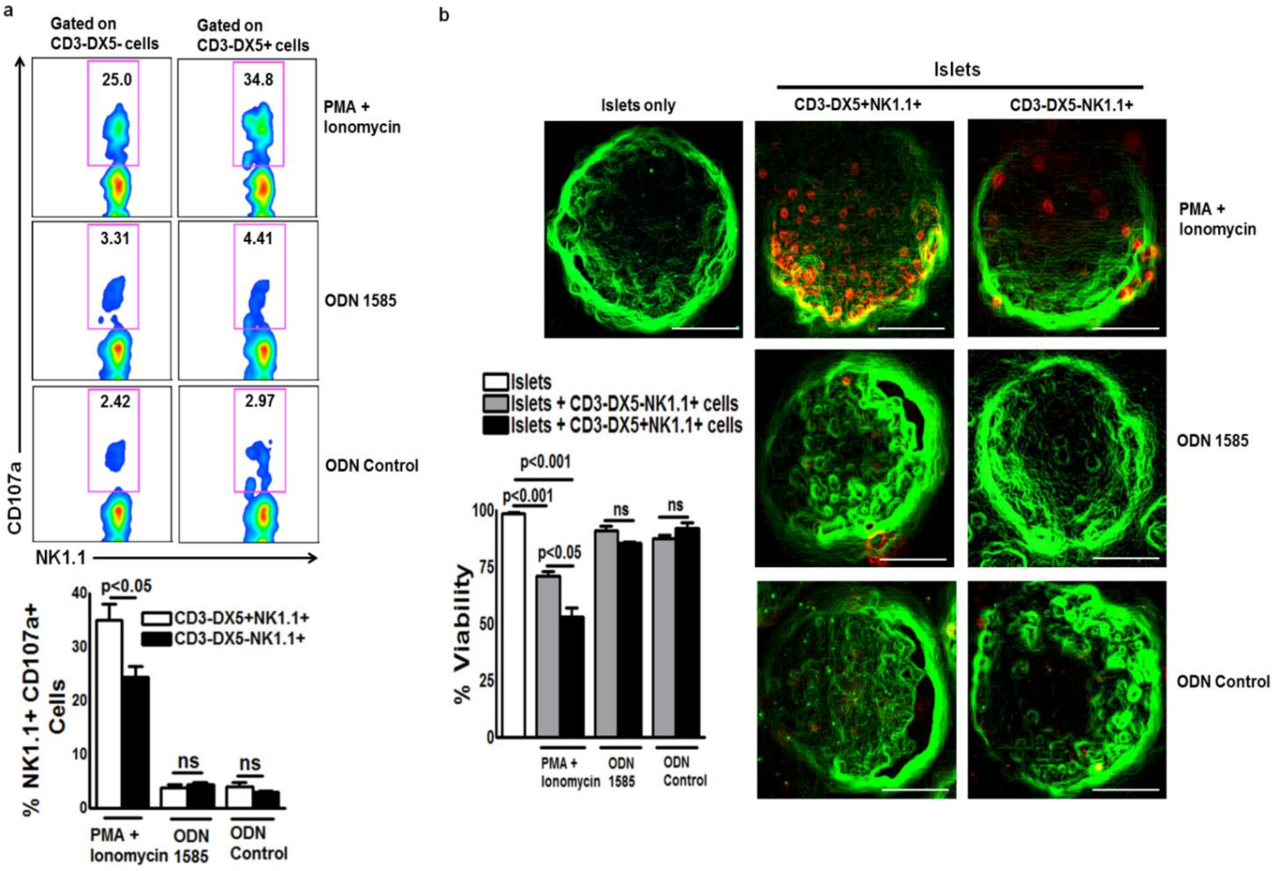

Supplementary Figure 9. The TLR9 agonist ODN 1585 immunization of T1D mice has no effect on NK cell cytotoxicity or islet viability. (a) C57BL/6 T1D mice were immunized intravenously with the TLR9 agonist ODN 1585 or were treated with control ODN (20 μg/mouse). After 30 days, liver cells were isolated and cultured with islets obtained from BALB/c mice in the presence of ODN 1585 or control ODN or PMA and ionomycin (20 μg/ml). CD3-DX5+NK1.1+ and CD3-DX5-NK1.1+ cell cytotoxicity was determined by flow cytometry using anti-CD107a antibody. Bar graphs show the means ± s.d. P values were generated by independent t-test. **(b)** CD3-DX5+NK1.1+ or CD3-DX5-NK1.1+ cells were isolated and cultured with BALB/c pancreatic islets at a ratio of 1,000:1 (1,000 CD3-DX5+NK1.1+ or CD3-

DX5-NK1.1+ cells and one pancreatic islet). The islets were labeled with CFDA/PI, and the percentages of viable islets were determined using a fluorescence microscope. A fluorescence microscopy figure is shown. Photographs are representative of staining patterns. Magnification 40X; scale bar = 50 μm (Right). Bar graphs show the means ± s.d. P values were generated by independent t-test. The data shown are representative of three independent experiments.

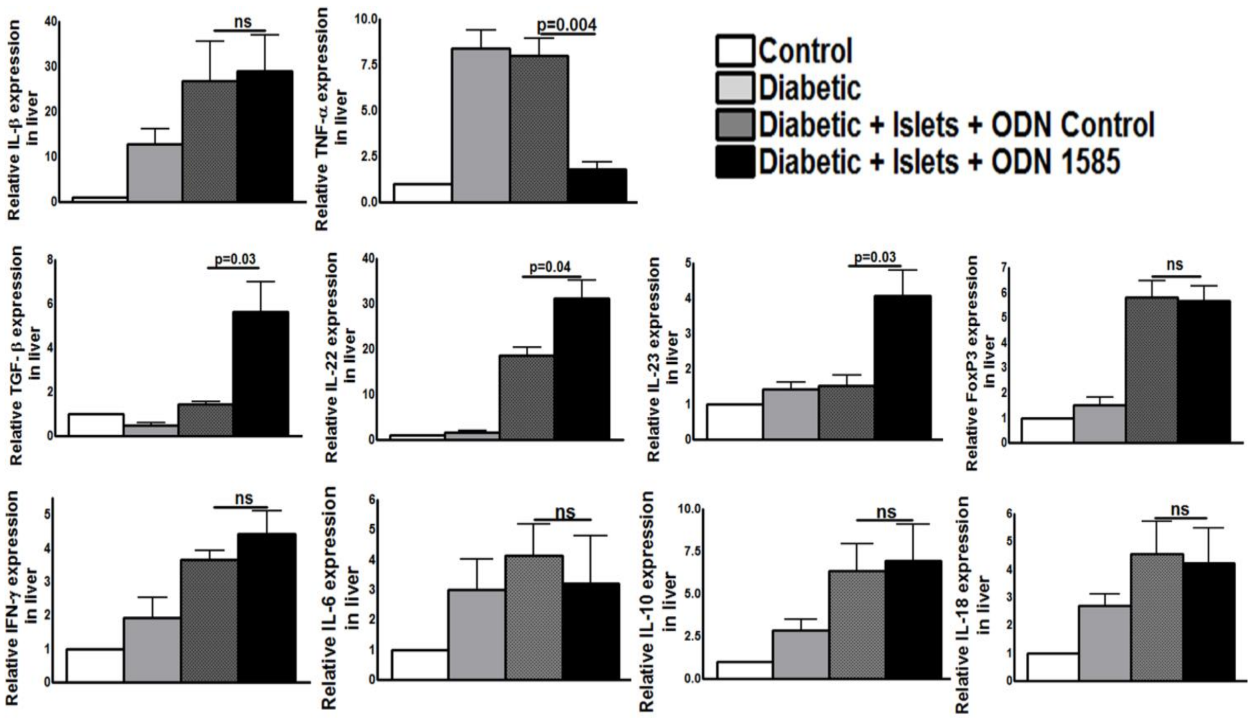

Supplementary Figure 10. C57BL/6 T1D mice were immunized intravenously with the TLR9 agonist ODN 1585 or treated with ODN control (20 µg/mouse). Thirty days after immunization, pancreatic islets from BALB/c mice were transplanted into the liver parenchyma of T1D C57BL/6 mice (recipients) as mentioned in Fig. 1. After 20 days, liver lymphocytes were isolated, and cytokine mRNA expression was determined by real-time PCR. Bar graphs show the means ± s.d. P values were generated by one-way analysis of variance (ANOVA). The data shown are representative of three independent experiments.

Supplementary Table 1: List of primers used in this study

S.No	Gene Name	Mouse Primer Sequences
1	IFN-γ	Forward: TCA AGT GGC ATA GAT GTG GAA GAA<br>Reverse: TGG CTC TGC AGG ATT TTC CAT G
2	TNF-α	Forward: CAT CTT CTC AAA ATT CGA GTG ACA A<br>Reverse: TGG GAG TAG ACA AGG TAC AAC CC
3	IL-18	Forward: ACT GTA CAA CCG CAG TAA TAC GC<br>Reverse: AGT GAA CAT TAC AGA TTT ATC CC
4	IL-6	Forward: TAC CAC TTC ACA AGT CGG AGG C<br>Reverse: CTG CAA GTG CAT CAT CGT TGT TC
6	IL-23	Forward: CAT GCT AGC CTG GAA GCG ACA T<br>Reverse: ACT GGC TGT TGT CCT TGA GTC C
7	IL-21	Forward: GCC TCC TGA TTA GAC TTC GTC AC<br>Reverse: CAG GCA AAA GCT GCA TGC TCA C
8	IL-1β	Forward: CAA CCA ACA AGT GAT ATT CTC CAT G<br>Reverse: GAT CCA CAC TCT CCA GCT
9	IL-22	Forward: CAT GCA GGA GGT GGT GCC TT<br>Reverse: CAG ACG CAA GCA TTT CTC AG
10	TGF-β	Forward: GGA TAC CAA CTA TTG CTT CAG CTC C<br>Reverse: AGG CTC CAA TAT TAG GGG CAG GGT C
11	IL-10	Forward: GGT TGC CAA GCC TTA TCG GA<br>Reverse: ACC TGC TCC ACT GCC TTG CT
12	FoXP3	Forward: CCT GGT TGT GAG AAG GTC TTC G<br>Reverse: TGC TCC AGA GAC TGC ACC ACT T
13	B-Actin	Forward: CTC TGG CTC CTA GCA CCA TGA AGA<br>Reverse: GTA AAA GAC AGC TCA GTA ACA GTC CG
14	TXNIP	Forward: CAG CCA ACT CAA GAG GCA AA<br>Reverse: ATT GGC AGC AGG TCT GGT CT
15	PDX1	Forward: TCA ACA GCT GCG ATC AGT A<br>Reverse: AAC ATCA CTG CCA GCT CCA CC
16	IRE1	Forward: GAA GAC GTC ATT GCA CGT GAA TT<br>Reverse: AGG TCC TGA ATT TAC GCA GGT
17	Foxa2	Forward: CAA GGA TGC CTC TCC ACA CTT<br>Reverse: TGA CCA TGA TGG CTC TCT GAA
18	Reg1a	Forward: TCC TGC TTA GCT CCA GGG AT<br>Reverse: TGA CCC TCC TGG CTT TAG GA
19	Reg2	Forward: GGA GCA GTG GCT CCC TAT TT<br>Reverse: TAT CAG CAG GCC CAG GTA CA
20	Reg3a	Forward: GCA TGC CCC TCT TCT CAC AT<br>Reverse: CCC CCA TGT TCA CCA GTG TT
21	Reg3g	Forward: GGA GCA GTG GCT CCC TAT TT<br>Reverse: TAT CAG CAG GCC CAG GTA CA
